# Replication, Gene Expression and Particle Production by a Consensus Merkel Cell Polyomavirus (MCPyV) Genome

**DOI:** 10.1371/journal.pone.0029112

**Published:** 2011-12-27

**Authors:** Friederike Neumann, Sophie Borchert, Claudia Schmidt, Rudolph Reimer, Heinrich Hohenberg, Nicole Fischer, Adam Grundhoff

**Affiliations:** 1 Institute for Microbiology and Virology, University Medical Center Eppendorf, Hamburg, Germany; 2 Heinrich-Pette-Institute, Leibniz Institute for Experimental Virology, Hamburg, Germany; Kantonal Hospital St. Gallen, Switzerland

## Abstract

Merkel Cell Polyomavirus (MCPyV) genomes are clonally integrated in tumor tissues of approximately 85% of all Merkel cell carcinoma (MCC) cases, a highly aggressive tumor of the skin which predominantly afflicts elderly and immunosuppressed patients. All integrated viral genomes recovered from MCC tissue or MCC cell lines harbor signature mutations in the early gene transcript encoding for the large T-Antigen (LT-Ag). These mutations selectively abrogate the ability of LT-Ag to support viral replication while still maintaining its Rb-binding activity, suggesting a continuous requirement for LT-Ag mediated cell cycle deregulation during MCC pathogenesis. To gain a better understanding of MCPyV biology, in vitro MCPyV replication systems are required. We have generated a synthetic MCPyV genomic clone (MCVSyn) based on the consensus sequence of MCC-derived sequences deposited in the NCBI database. Here, we demonstrate that transfection of recircularized MCVSyn DNA into some human cell lines recapitulates efficient replication of the viral genome, early and late gene expression together with virus particle formation. However, serial transmission of infectious virus was not observed. This in vitro culturing system allows the study of viral replication and will facilitate the molecular dissection of important aspects of the MCPyV lifecycle.

## Introduction

Merkel cell polyomavirus (MCPyV) is one of nine human polyomaviruses known to date [1]–[8]; out of these, four (JC, BK, TSV and MCPyV) are known to induce severe disease in immunosuppressed patients. MCPyV was identified in 2008 in primary tumor material from Merkel cell carcinomas (MCC) [2]. Subsequently, up to 85% of all MCC cases were found to carry the viral DNA monoclonally integrated in the tumor cells [9]–[13]. The high frequency of MCPyV detection in MCC tissue, the monoclonal integration patterns [2], [13], and the observation that the tumor cells constitutively express viral T-antigens strongly support the classification of MCPyV as a human tumor virus [14].

As in all polyomaviruses, the early region of the MCPyV genome encodes large and small T Antigens (LT- and ST-Ag, respectively). These genes are transcribed immediately upon nuclear delivery of the viral genome and drive the cell into S-phase, thus creating conditions which are favourable for viral DNA replication [15]. LT-Ag furthermore exhibits helicase activity and recruits cellular replication factors to the viral episome, functions which are of crucial importance for replication of viral DNA [15]. In addition to LT- and ST-Ag, the early region of MCPyV also encodes a 57 KDa protein of unknown function, as well as a viral miRNA that is located in antisense orientation to the LT-gene; as in other polyomaviruses, this miRNA may serve to downregulate LT-transcripts during the late stages of the infection cycle [16].

Strikingly, viral genomes isolated from MCC material unequivocally carry frameshift or STOP codon mutations within the LT-Ag coding region, resulting in the expression of truncated LT proteins that lack the C-terminal origin binding and ATPase/Helicase domains, and which are thus unable to support viral replication [13], [17]. All MCC-derived LT-Ags, however, maintain the Rb binding domain, indicating that there is selective pressure to preserve Rb-inactivating functions during MCC pathogenesis. At present, when and exactly why viral genomes in MCC acquire truncating mutations is unclear.

The late gene region of polyomviruses encodes structural proteins, the major capsid protein VP1 and the minor capsid proteins VP2 and VP3. All three open reading frames are present in the MCPyV genome, although VP3 may in fact not be functional [18]. No agnoprotein has been described for MCPyV so far [2], [19]. Polyomavirus late gene transcription usually is initiated concomitantly with viral DNA replication [20], [21]. In some members of the polyomavirus family, LT-Ag regulates transcription of the structural proteins by binding to the late promoter region [15]. In SV40, late transcription is independent of viral replication [21], [22]. Polyomavirus assembly and in particular egress of viral particles from the cell are, despite of extensive studies in the prototypic SV40 prototype, still incompletely understood processes: for example, although induction of cell lysis is thought to be the primary mechanism of virion release, accumulation of viral particles in cytoplasmic compartments with subsequent shedding from intact cells has also been reported [23], [24].

To date, the natural host cell of MCPyV remains elusive; although MCPyV DNA can be amplified from skin swabs of healthy individuals, the amount of DNA recovered is generally rather low [7], [25], and it is therefore unclear whether skin cells represents the primary reservoir for MCPyV infection. In contrast to the often broad tissue distribution of polyomavirus receptors, viral replication and production of infectious progeny is usually highly restricted and occurs only in few cell types *in vitro* and *in vivo*. As a consequence, the reconstitution of *in vitro* polyomavirus replication systems is often challenging. For example, the available *in vitro* systems to study BK virus and JC virus infection [26] still only support some aspects of the viral life cycle, and to date no replication systems has been established for WU and KI virus [1], [4]. To begin addressing the question of cell type specificity of MCPyV replication, and to generate a system that allows the study of the MCPyV lifecycle, we constructed a synthetic consensus MCPyV genome (MCVSyn) based on all MCPyV sequences available in the NCBI database as of June 2008. Using this clone, we demonstrate robust and reproducible viral DNA replication in 3 out of 17 human cell lines tested. Replication can be significantly enhanced by co-expression of the early region *in trans*. Furthermore, we observe early and late gene transcription in these three cell lines, and detect electron-dense particles with a size of 40–50 µm in cells transfected with viral DNA. Despite of the above, however, we did not observe serial transmission of MCPyV infection, a finding which is in line with a recent report that has used an approach similar to ours [27]. We also provide evidence that MCPyV particles differ from SV40 virus particles with regard to shape and size, and, using confocal microscopy, demonstrate that VP1 localization significantly differs between MCPyV- and SV40-transfected cells. Thus, in addition to their overall low abundance, low infectivity of MCPyV particles may be another factor that hampers transmission.

## Results

### Construction of a synthetic consensus MCPyV genome (MCVSyn)

The goal of this study was to investigate viral replication in the context of a full length viral genome. However, at the time when our study was initiated, no field isolates of MCPyV had been described, and the only available sequences were derived from the integrated, defective viral genomes in MCC. When we aligned theses sequences, we observed that, besides of the mutations which truncate the LT-Ag ORF, a number of additional sequence variations were distributed throughout the viral genome. Some of these variations are likely to represent nucleotide polymorphisms already present in the parental MCPyV strain that had originally infected the cell giving rise to the tumor. However, we could not rule out the possibility that, akin to the hallmark LT-Ag truncating mutations, some mutations may also be the result of selectional pressure to abrogate viral replication. Instead of repairing the STOP mutations in one of the MCC-derived clones, we therefore opted to generate a synthetic viral genome (termed MCVSyn) that represented the consensus of all MCC-derived MCPyV sequences, reasoning that such a consensus should come closest to a wild type MCPyV genome. Accordingly, we aligned all MCPyV sequences deposited in the NCBI database as of June 2008, and synthetically constructed the full length genome in two fragments (nts. 1044-4034 and 4003-1056, respectively) in a bacterial vector backbone. The fragments were subsequently joined to produce pMCVSyn, a construct from which the full-length circular viral genome can be produced by restriction digestion and subsequent intramolecular ligation (see Figure S1 and Material & Methods for details). The sequences of pMCVSyn and MCVSyn have been deposited in GenBank under accession numbers JN707598 and JN707599, respectively.

**Figure 1:**
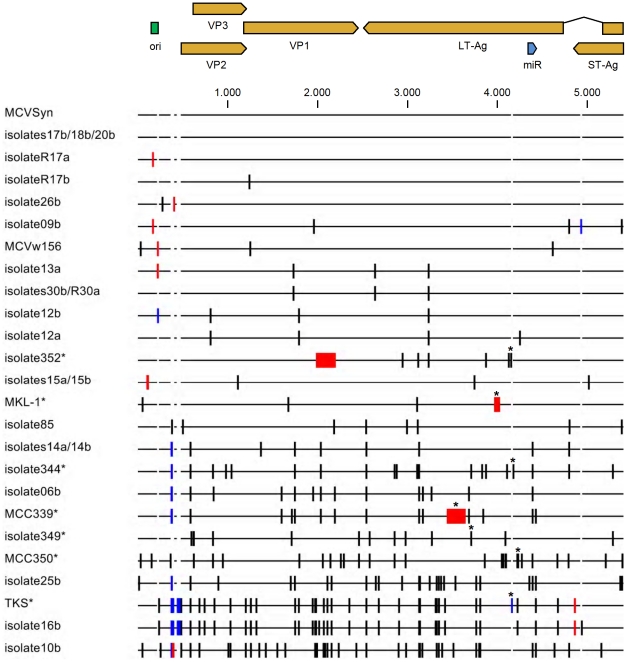
Alignment of MCVSyn and full length MCPyV sequences. The illustration is based on a multiple sequence alignment using the Clustal W algorithm (see Table S3 for accession numbers used in the alignment) of MCVSyn and all full length MCPyV sequences deposited in the NCBI Database as of August 2011. Aligned genomes were compared to the consensus sequence (which is identical to MCVSyn as well as the isolates 17b, 18b and 20b). Nucleotide substitutions/mismatches relative to this sequence are shown as vertical black bars, whereas deletions are shown in red. Nucleotide insertions in a given sequence are shown as blue bars, and register as gaps in the backbone of the remaining genomes. Genomes that were isolated from MCC or MCC-derived cell lines are marked by an asterisk; the mutations which lead to the truncation of LT-Ag sequences in these genomes are likewise marked.

While our study was in progress, Schowalter et al. [7] reported the isolation of 20 full-length MCPyV sequences from skin swabs of healthy individuals. Indeed, our MCC-derived consensus genome is identical to the field strain consensus sequence, which is furthermore perfectly preserved in the three individual isolates 17b, 18b and 20b (acc. numbers HM011549, HM011550 and HM011551, respectively). MCVSyn displays one silent nucleotide exchange in the VP1-coding region when compared to R17b (Table S1), whereas in R17a, a single nucleotide deletion can be found in the early palindrome region of the NCCR. An alignment of the MCVSyn sequence and seven tumor-derived genomes bearing LT-Ag mutations as well as 22 non-MCC isolates, including the 20 field isolates described in [7], is shown in Figure 1 (see Figure S2 for a phylogenetic tree based on these alignments).

### Early gene expression in SV40 and MCVSyn transfected cells

**Figure 2:**
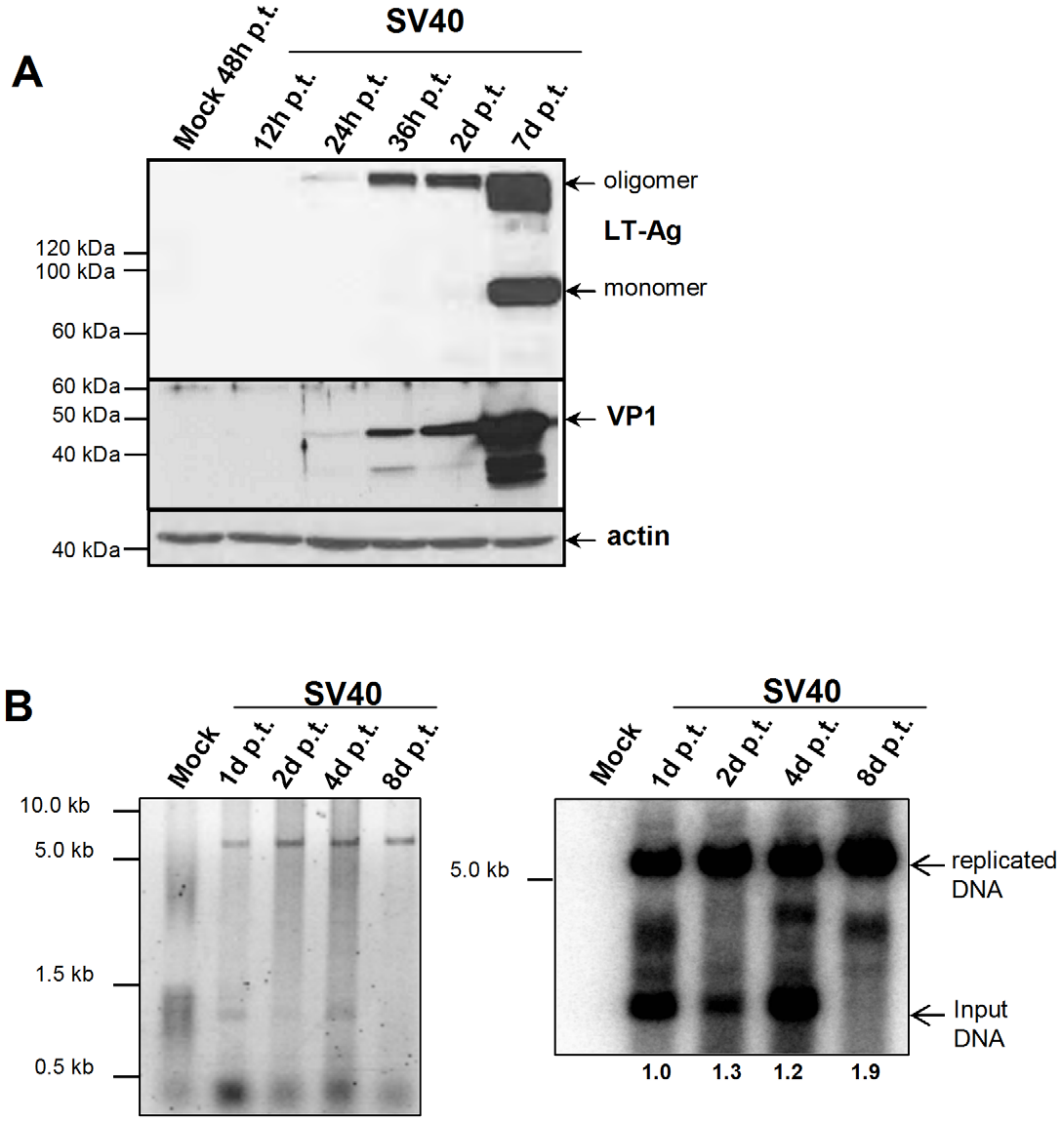
SV40 replication and gene expression in CV-1 cells transfected with SV40 DNA. (A) 100 ng of intramolecular religated SV40 viral DNA was transfected in CV-1 cells and cells were lysed 12 h, 24 h, 36 h, 2d and 7d post transfection. Protein lysates were subsequently analyzed for SV40 LT-Ag (Pab419 antibody) and VP1 expression (α-VP1 polyclonal rabbit serum) by SDS-page and Western Blotting. Staining of actin was used to ensure that equal protein amounts were loaded per lane. (B) Low molecular weight DNA was isolated from SV40 DNA transfected CV-1 cells at the indicated time points by HIRT extraction, 1 µg DNA was DpnI and EcoRI digested; DNA was separated on an agarose gel and stained with EtBr (left panel), followed by southern blotting and detection of viral DNA using a ^32^PdCTP-labeled SV40 LT-Ag PCR fragment as a probe. The blot was exposed for 30 min. Numbers below the lanes correspond to the quantification of newly replicated DNA using a Fuji phosphoimager FLA7000 and MultiGauge software.

In order to investigate early gene expression by MCVSyn genomes, we transfected re-circularized episomes into 12 established human cell lines, 3 primary human cell types, 3 african green monkey cell lines as well as 3 rodent cell lines (Table 1), followed by RT-PCR and/or western blotting to detect early and late gene products at various time points post transfection. To provide an internal control, we performed all assays in parallel with circularized SV40 DNA transfected into CV-1 cells, a cell line that is known to be fully permissive for SV40 replication and virion production. As shown in Figure 2A, SV40 LT-Ag (SDS-resistant LT-oligomers and monomers as described before [28]) as well as VP1 protein expression was readily detectable as early as 24 h post transfection, with both proteins accumulating to high levels over the following 6 days. Between 4 and 6 days post transfection, the cultures consistently displayed morphological changes indicative of early cytopathic effects (i.e., the appearance of rounded and enlarged cells); all SV40-transfected CV-1 cultures were completely lysed by day 11 (see Figure S3).

**Table 1:** Cell lines used to study MCVSyn replication, early and late transcription as well as particle formation.

Cell line	Species	DNA replication	LT-Ag protein expression^a^	LT-Ag, VP1 transcripts^b^	Transfection-Efficiency(%)^c^
CV-1	african green monkey	−	−	n.t.	∼40
Vero	african green monkey	−	−	n.t.	∼40
COS-7	african green monkey			n.t.	∼20
N2A	mouse	−	−	n.t.	∼40
NIH3T3	mouse	−	−	n.t.	∼10
pBRK	rat	−	−	n.t.	∼5
**293**	human	**+**	**+**	**+++**	∼60
293T	human	−	−	n.t.	∼60
HeLa	human	−	−	n.t.	∼40
A549	human	(+)	−	n.t.	∼40
Bea 2B	human	−	−	n.t.	∼20
**H1299**	human	**++**	**++**	**++**	∼40
Saos-2	human	(+)	−	n.t.	∼10
HaCaT	human	−	−	n.t.	∼10
Primary keratinocytes	human	−	−	n.t.	∼5
NHDF	human	(+)	−	n.t.	∼5
Huvec	human	(+)	−	n.t.	∼10
**PFSK-1**	human	**+++**	**+++**	**++**	∼40
LAN-1	human	−	−	n.t.	∼10
IMR-32	human	−	−	n.t.	∼10
Sy5y	human	−	−	n.t.	∼5
**293 MCPyV LT**	human	**++++**	n.t.	n.t.	∼60

a : LT-Ag protein expression was tested by Immunoblotting 2d, 4d, 8d, 12d, 44d past transfection;

b : transcripts were tested at 2d, 4d, 8d and 12d p.t. using quantitative realtime PCR and normalized to GAPDH. n.t.: not tested.

c : Transfection efficiency was determined for each cell line using 100 ng GFP expression construct in 10^5^ cells, 48 h p.t cells were analysed by FACS for GFP expression.

**Figure 3:**
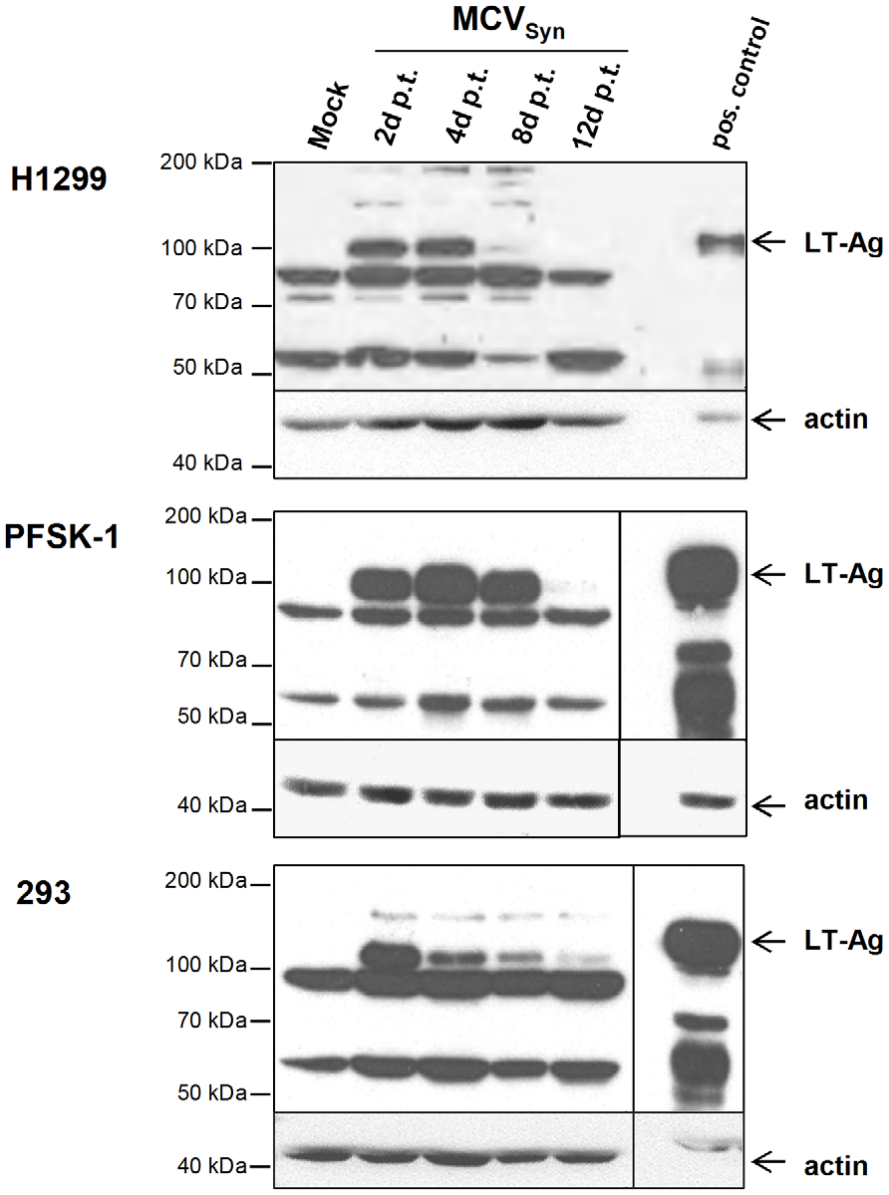
LT-Ag expression in human cell lines transfected with MCVSyn DNA. 5×10^4^ H1299, PSFK-1 or 293 cells were transfected with 100 ng re-circularized MCVSyn DNA or equivalent amounts of pUC18 DNA (Mock control). At the indicated time points, cells were lysed and analyzed by immunoblotting for MCPyV LT-Ag expression using the monoclonal LT-Ag antibody Cm2B4. Equal protein loading was confirmed by re-incubating the membrane with an anti-actin antibody. An LT-Ag expression control (pos. control; transient transfection with a CMV-promoter driven LT-Ag expression construct for 48 h) was loaded as an internal control.

As shown in Table 1, out of the 21 cell lines and primary cell cultures that had been transfected with MCVSyn DNA, only three lines consistently showed strong LT-Ag expression when transfected with re-circularized MCVSyn DNA. The protein was most efficiently expressed in the primitive neuroectodermal tumor cell line PFSK-I, followed by the human non-small cell lung carcinoma cell line H1299 and human embryonic kidney 293 cells. However, as shown in Figure 3, in contrast to the accumulation of LT-Ag protein in SV40-transfected CV-1 cells, protein expression by MCVSyn reached a peak between 2 and 4 days post transfection, and thereafter declined to levels which were barely detectable at day 12.

These results suggest that early gene expression in the context of the full-length MCVSyn episome is efficient in some, but not all of the tested human cell lines or primary cell cultures. However, since none of the LT-Ag expressing cells consistently showed a CPE, and since furthermore LT-Ag expression declined after reaching an initial peak, we suspected that the virus was unlikely to spread through the cultures; this could have been due to inefficient viral DNA replication, a block in the production of mature viral particles, non-permissiveness of the tested cells for viral entry (e.g. due to the lack of the authentic entry receptor), or a combination of the above.

### DNA replication of MCVSyn is efficient in 293, H1299 and PFSK-1 cells

We monitored the efficiency of viral DNA replication in cells transfected with 100 ng of re-circularized viral genomes at two, four, eight or twelve days post transfection by isolating episomal DNA using the HIRT extraction protocol. The DNA was subsequently digested with *EcoRI* as well as *DpnI* and analyzed by Southern Blotting to detect replicated genomes. In this assay, non-replicated input plasmids are digested by the restriction enzyme *DpnI*, which cleaves methylated *dam* sites. As eukaryotic cells lack the bacterial *dam* methyltransferase, replicated episomes become resistant to *DpnI* cleavage. As shown in Figure 2B, significant amounts of such replicated episomes could already be detected after one day of transfection of SV40 DNA into CV-1 cells. A modest increase of replicated products was observed over time, such that at day 8 levels were approximately 2fold higher than on day 1.

**Figure 4:**
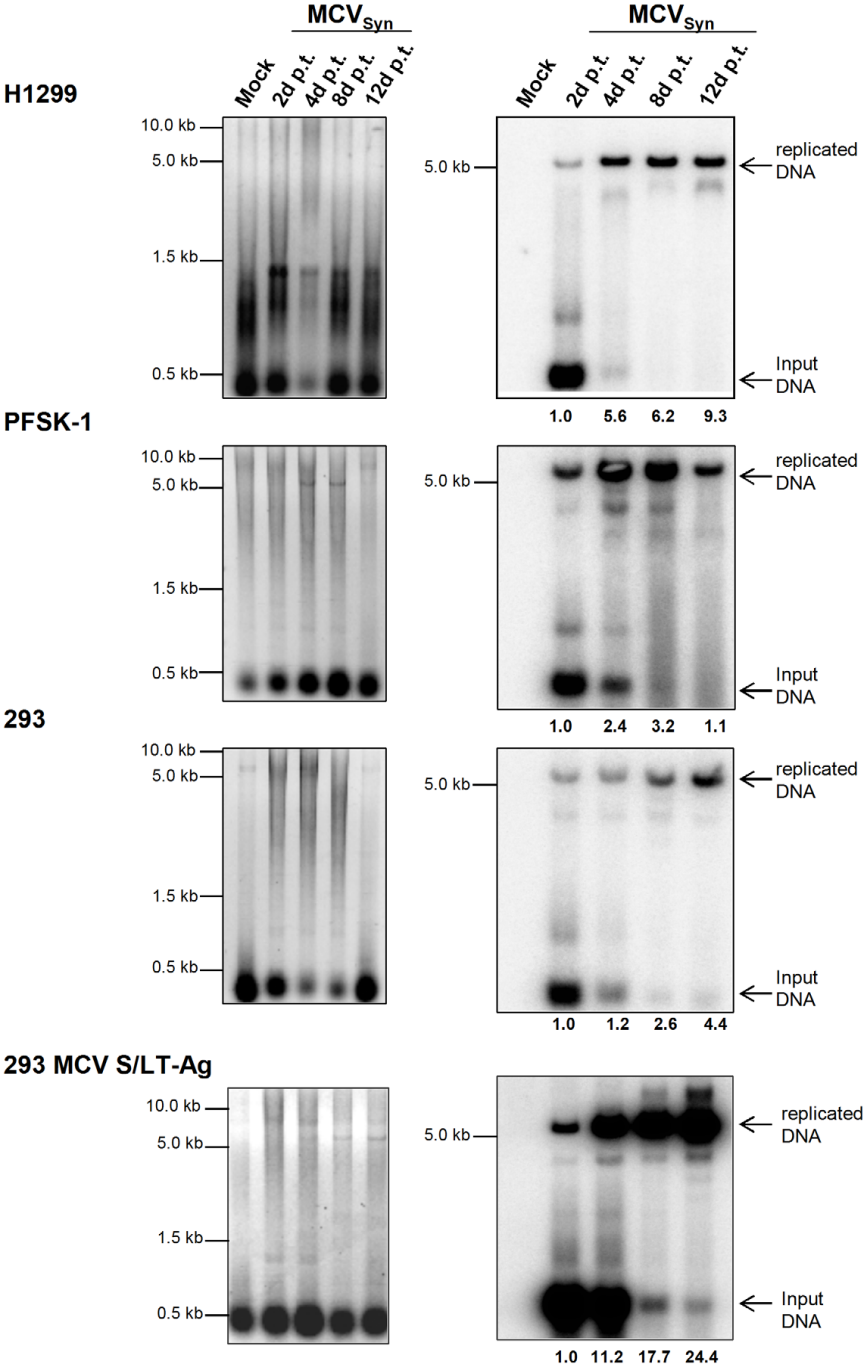
MCVSyn replication assays in H1299, PFSK-1 and 293 cells. 5 µg low molecular weight DNA isolated from cell cultures at the indicated time points post-transfection with MCVSyn DNA was digested with DpnI and EcoRI, separated on an agarose gel and stained with EtBr (left panels), then transferred via southern blot and probed with a radioactively labelled LT-Ag PCR fragment [13]. The blot was exposed for 24 h and scanned using a Fuji phosphoimager FLA7000; MultiGauge software was used for quantification.

**Figure 5:**
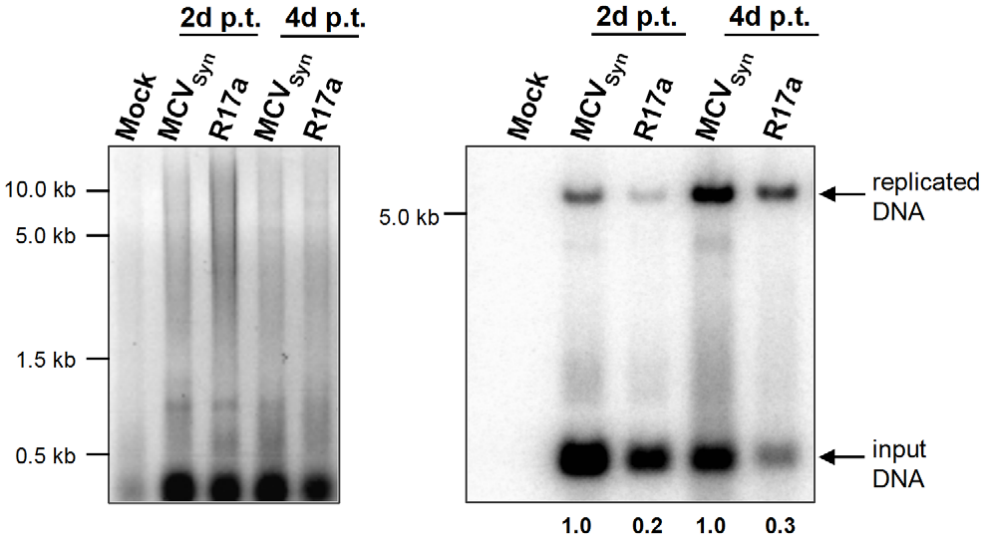
R17a replication in PFSK-1 cells. 2 µg low molecular weight DNA from PFSK-1 cells transfected with MCVSyn or R17a viral DNA was EcoRI and DpnI digested and separated on an agarose gel, followed by EtBr staining (left panel). The DNA was transferred via southern blotting and probed with a radioactively labelled LT-Ag PCR fragment (right panel). The blot was exposed for 24 h.

In the three cell lines that had displayed robust LT-Ag after transfection of MCVSyn genomes, we were also able to readily detect replicated episomes, albeit with different dynamics and generally at lower levels than in to SV40 transfected CV-1 cells. In Figure 4, we show a representative experiment in which transfection efficiencies between the individual cell lines were similar (30%; 32% and 40% for H1299, PFSK-1 and 293, respectively). In all three cell lines, viral DNA replication products were observed already at the earliest tested time point (i.e., day 2). However, whereas PFSK-1 cells showed a marked peak of replication products between four and eight days post transfection, levels in H1299 and 293 cells continuously increased over the entire twelve day time course. In H1299 cells, the increase was more rapid than in 293 cells, reaching final levels of 9.3fold at day 12 (relative to the levels observed at day 2) of the experiment. Despite of the continuous increase of relative levels observed in H1299 and 293 cells, however, we consistently observed the strongest absolute signal intensities in PFSK-1 cells, even though the peak is only transient. Nonetheless, neither PFSK-1 nor 293 or H1299 cells showed a CPE, and we were unable to detect LT-Ag or VP1 expression at late time points of infection (48 days) in these cultures (data not shown). Interestingly, as shown in Figure 5, the MCPyV field isolate R17a [7], which differs only by a single nucleotide deletion in the non-coding palindrome region of the MCPyV core replication origin ([29], see Figure 1 and Table S1) reproducibly exhibited an approximately 5 fold lower replication efficiency in PFSK-1 cells when compared to the MCVSyn genome.

Although most of the other 18 human, simian or rodent cell cultures listed in Table 1 scored negative in the above replication assay, we additionally observed weak and/or transient replication in the two primary cell cultures (human umbilical vein-derived endothelial cells/HUVEC and normal human dermal fibroblasts/NHDF) as well as the osteosarcoma-derived cell line Saos-2 (Figure S4). Since we had been unable to detect LT-Ag protein in these cells in our western blots (see Table 1), this observation demonstrates that low LT-Ag levels that are below our limit of detection are sufficient to support DNA replication, and furthermore may explain why we are able to observe continued DNA replication in 293 and H1299 at late time points post transfection, even though the LT-Ag levels in these cultures are low (Figure 3).

### Constitutive expression of the MCPyV early gene locus *in trans* increases viral replication in 293 cells

Given the decline of LT-Ag levels in H2199 and 293 cells over time, we wondered whether stable provision of LT-Ag *in trans* may lead to a further increase in DNA replication efficiency, which in turn could allow the effective production of infectious viral progeny. Accordingly, we generated 293 cells which stably express the early region of MCPyV (i.e. ST- as well as LT-Ag encoding sequences) under the control of the CMV promoter. As shown in the lower panel of Figure 4, after transfection of re-circularized MCVSyn DNA these cells showed pronounced and continued amplification of viral replicons, such that replicated episomes at 4 and 12 days post transfection were approximately 11 or 24 times, respectively, more abundant than on day 2. Despite of this effective DNA replication, however, we did not observe a CPE or (as discussed later) production of infectious supernatants in these cells.

### Late gene transcription and VP1 expression in MCVSyn-transfected cells

**Figure 6:**
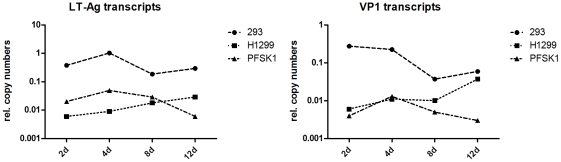
Real-time PCR to quantify early and late gene transcripts in cells transfected with MCVSyn. RNA was isolated at the indicated time points after transfection, DNAse I digested and used for cDNA synthesis followed by real time PCR using a LT-Ag or VP1 specific primer set. Results were normalized against GAPDH transcript levels.

Given our inability to observe a classical CPE and/or efficient horizontal spread of viral infection in the tested cell cultures, we next wondered whether late gene expression occurs in these cells. We first analyzed VP1 as well as LT-Ag transcription at different time points by performing quantitative realtime RT-PCR. As shown in Figure 6, LT and VP1 transcripts were readily detectable at day 2 in 293, H1299 as well as PFSK-1 cells. The cell lines differ in the amount of transcripts produced, with 293 cells showing an approximately 10fold higher transcript level at 4 days post transfection. These results demonstrate that late gene transcription does occur, but they do not exclude the possibility that late gene products are not efficiently translated, fail to localize in appropriate sub-nuclear compartments or do not accumulate to levels that allow virion production. We therefore analyzed VP1 protein expression and localization. We performed wide field as well as confocal microscopy immunofluorescence analysis (IFA) using a polyclonal VP1 serum that is suitable to detect MCPyV VP1 in IFA and immunohistochemistry, kindly provided by Christopher Buck (NIH) [18], [19]. As before, experiments were performed in parallel with SV40 transfected CV-1 cells in order to directly compare VP1 protein expression and localization.

**Figure 7:**
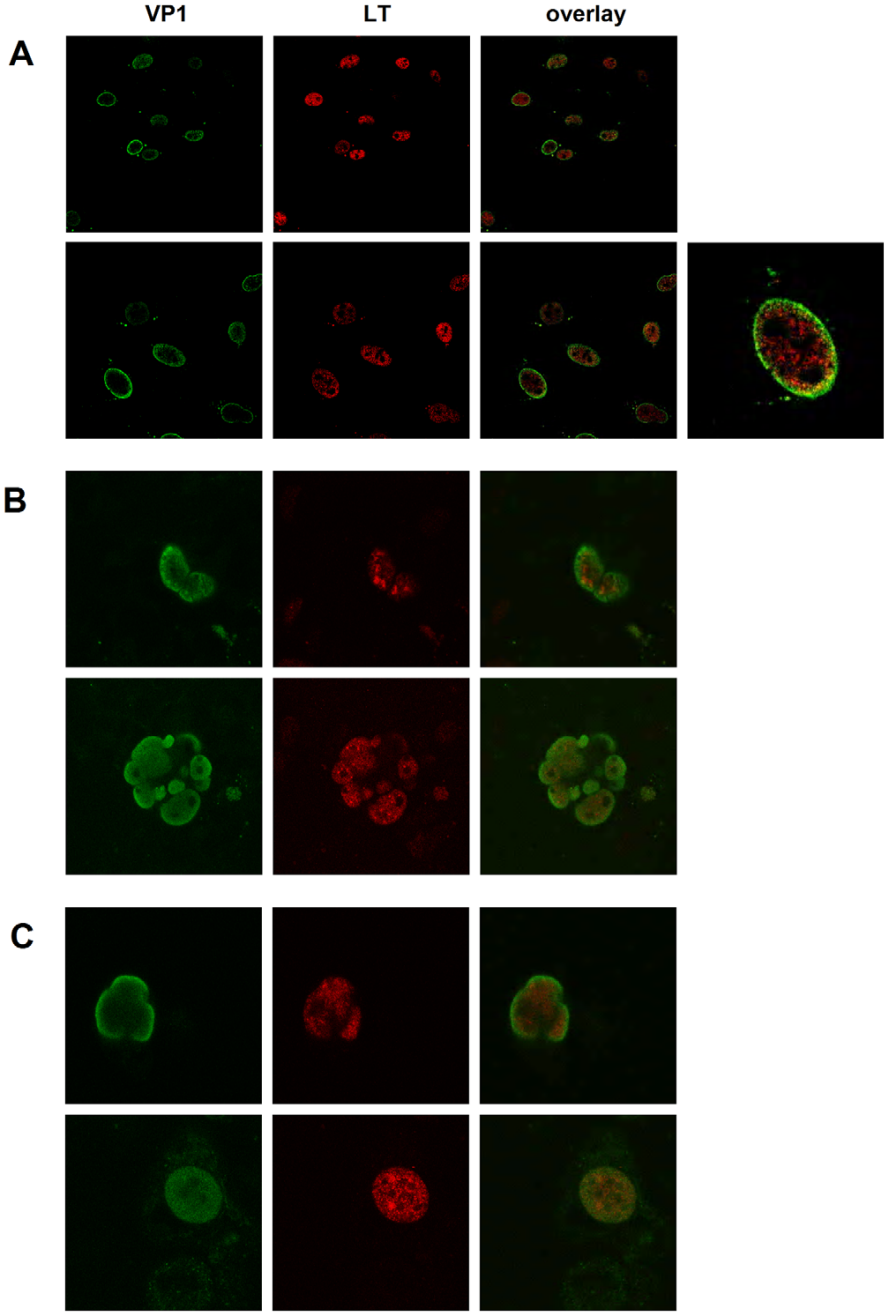
Subcellular localization of LT-Ag and VP1 protein in cells transfected with religated viral DNA. (A) Double staining of CV-1 cells transfected with SV40 viral DNA. 4d p.t. the cells were fixed, and VP1 was detected with a polyclonal anti-VP1 antibody. LT-Ag was visualized with the monoclonal anti-LT antibody Pab419. Z-stack pictures were taken using confocal microscopy. Each picture represents an individual Z-stack. VP1 staining was observed primarily in speckles close to or at the nuclear membrane. LT-Ag staining was observed throughout the nucleoplasm with the nucleoli excluded. In some cells granular LT-Ag staining was observed. The panel on the lower right represents a 3× zoomed picture of a CV1 transfected cell with the two channels merged. Double staining of Merkel cell polyomavirus VP1 and LT-Ag in H1299 cells (B) and PFSK-1 cells (C) 4d p.t. reveals inner peripheral nuclear localization of MCVSyn VP1 protein. VP1 was visualized with a polyclonal anti-VP1 serum and anti-rabbit FITC, while LT-Ag was visualized with the monoclonal antibody Cm2B4 specifically recognizing MCPyV LT-Ag. 40 Z-stack pictures were taken scanning through the cells using a 63× magnification and 2fold zoom on a confocal microscope. The picture shown represents an individual image from the center of a Z-stack.

As shown in Figure S5, at 48 h post transfection 50–60% of CV-1 cultures transfected with SV40 DNA cells expressed LT-Ag (given that the transfection efficiency in these experiments was approximately 60%, this indicates that LT-Ag expression is detectable in nearly 100% of the transfected cells). At the same time, approximately half of these cells already express VP1 protein (Figure S5A). In Figure 7A, we present a confocal analysis of the cells at 72 h post transfection. As expected LT-Ag, is strictly nuclear with the nucleoli being excluded. In the majority of cells LT-Ag was evenly distributed throughout the nucleus in a speckled pattern, likely to represent sites of DNA replication (center panels in Figure 7A). As shown in the left panels, in accord with previous reports we detect SV40 VP1 predominantly in speckles in the nuclear periphery or at the nuclear envelope, where viral particles are thought to assemble and later accumulate prior to the penetration of the nuclear membrane [30], [31], [32].

The percentage of MCVSyn-transfected H1299 and PFSK-1 cells that displayed high-level expression of early or late MCPyV antigens was significantly lower as in our SV40 experiments (approximately 5% at 35–40% transfection efficiency; see Figure S5 B). Similar to SV40 LT-Ag, the MCPyV LT-Ag localized to nuclear speckles in H1299 as well as PFSK-1 cells (center panels of Figure 7B and 7C, respectively). Although the MCPyV VP1 protein also shows strictly nuclear localization and predominantly localizes to perinuclear regions, we did not observe the distinct nuclear envelope localization seen in SV40 transfected CV-1 cells. Likewise, only a few VP1-expressing cells display accumulation of VP1 in speckles, similar to the cells shown in the upper left panel of Figure 7B. These findings suggest that LT-Ag and VP1 protein are correctly localized only in some MCVSyn-transfected H1299 and PFSK-1 cells. However, as such cells were nevertheless consistently detected, we hypothesized that at least a fraction of the transfected cells should support MCPyV replication as well as virus assembly, albeit the total amount of virion particles produced by the cultures was expected to be much lower than in SV40-transfected CV-1 cells.

### Detection of viral particles using density ultracentrifugation and electron microscopy

In order to detect virion particles produced by H1299 and PFSK1 cells, we performed Optiprep™ (Iodixanol) gradient ultracentrifugation as described before [18]. Again, SV40-transfected CV-1 cells were used as an internal standard. Fractions were analyzed by western blotting for 45/90 kDa VP1 monomer and dimers, serving as a marker for virion-containing fractions. Additionally, DNA isolated from all fractions was subjected to micrococcal nuclease digestion, followed by quantitative PCR using VP1 Primers.

**Figure 8:**
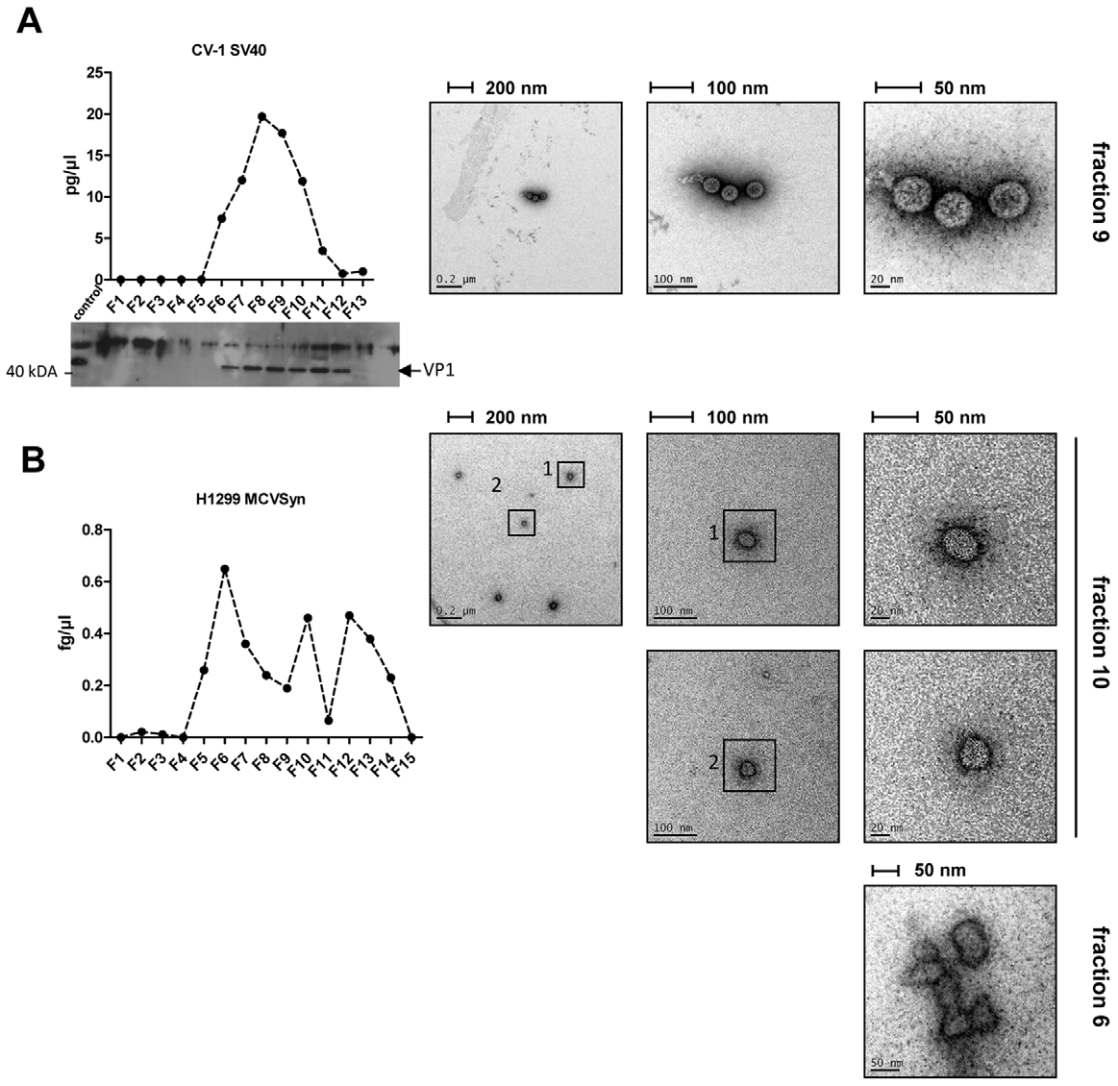
Density gradient centrifugation of SV40 and MCVSyn particles. Optiprep™ gradient centrifugation was performed with cell lysates from CV1 (A) and H1299 (B) cells 4d after transfection with viral DNA. 15×250 µl fractions were collected (fraction 1 represents the fraction with the highest density and fraction 15 represents the lowest density fraction). (A) Left panel: Real time PCR of micrococcal nuclease treated fractions was performed using SV40 VP1 primer sequences. 20 µl of each gradient fraction was loaded on a 10% SDS-page followed immunoblotting using anti-VP1 serum. Right panel: Negative EM staining of SV40 particles identified in fraction 9. (B) Left panel: Real time PCR results of H1299 MCVSyn gradient fractions after micrococcal nuclease treatment using MCPyV VP1-specific primers. Right panel: Negative EM staining of particles identified in fractions 10 and 6.

As shown in Figure 8A, western blot analysis o with a SV40 VP1-specific polyclonal serum indicated the presence of virion particles in fractions 6 to 12. The detection of VP1 perfectly correlates with the realtime PCR-based detection of genomic SV40 DNA in these fractions (Figure 8A). Negative staining of the peak fraction (fraction 9) readily identified virion particles of typical shape and size (approx. 45–50 nm; see right panels in Figure 8A) by electron microscopy (EM).

Micrococcal-nuclease resistant viral DNA, indicative of encapsidated viral DNA, was also observed in identically prepared gradient fractions from MCVSyn-transfected H1299 cells (Figure 8B). Similar results were observed in PFSK-1 cells (data not shown). However, consistent with the absence of cytopathic effects and lack of horizontal spread, the DNA amounts in these fractions were significantly (approx. 4–5 orders of magnitude) lower than in SV40-transfected CV-1 cells (see Figures 8B and 8A, respectively). The DNA was also more broadly distributed across the gradient fractions, with sub-peaks appearing in the high-molecular weight fraction 6, fraction 10 (which would be expected to contain viral particles based on the SV40 results), as well as the low molecular weight fraction 12. Indeed, as shown in the upper and middle rows of the right panel in Figure 8B, using negative EM staining we were able to detect particles that are likely to represent MCPyV virions in fraction 10 of our H1299 gradient preparations, although at much lower frequency than in SV40-transfected CV-1 cells. Overall, the particles also seemed to be somewhat smaller and of a slightly less regular/round shape than SV40 virions (compare upper right panels in Figure 8A and -B).

Isolated particles were absent from fraction 6, despite of the peak in viral DNA detected in these fractions. However, in accord with the higher molecular weight of these fractions we observed clusters of irregularly shaped, approximately 20–50 nm particles that may represent aggregates of misshaped viral particles. We were unable to detect any particles in the low molecular weight fraction 13, indicating that the DNA in this fraction is not virion-associated, but may rather be embedded in and protected by sub-particle sized cellular debris or membrane structures.

### Localization of electron-dense particles in cells transfected with MCVSyn

**Figure 9:**
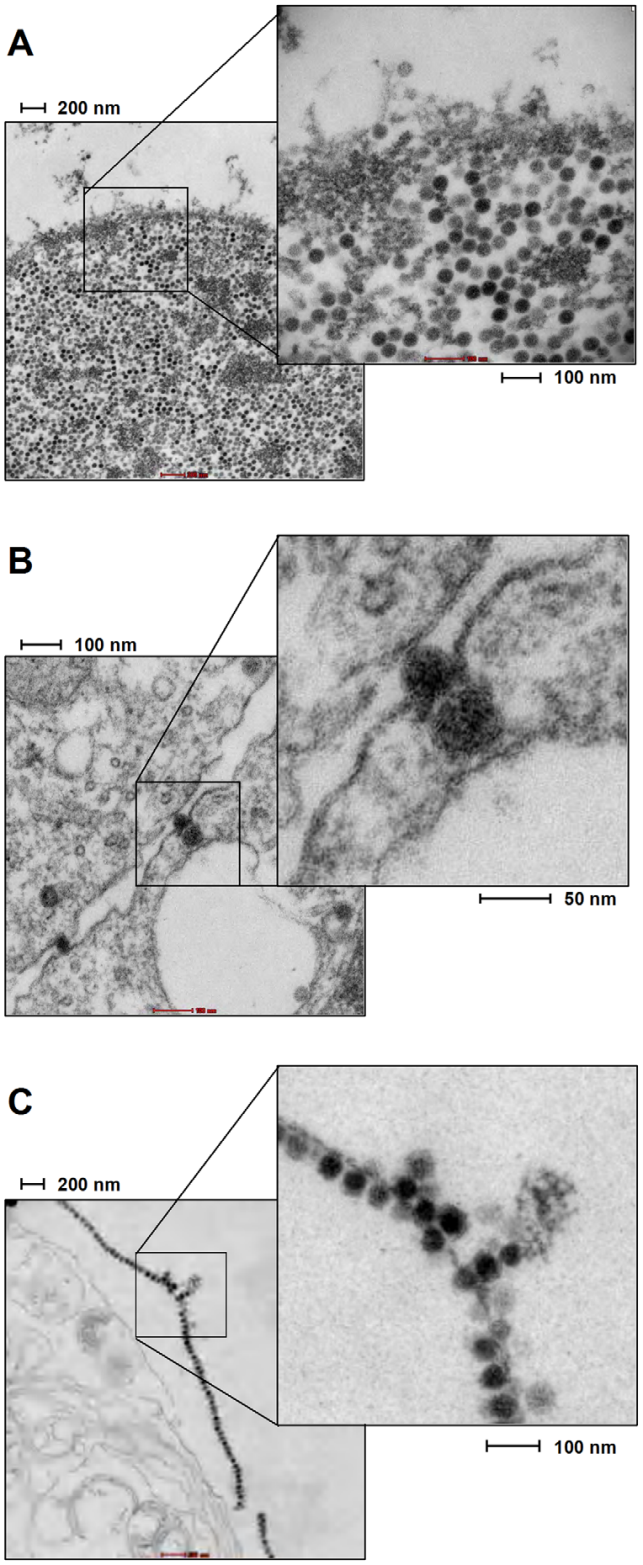
Electron micrographs of viral particles in SV40-transfected CV-1 cells. Images were prepared from CV-1 cultures at 4 days post transfection with SV40 DNA. (A) Accumulation of SV40 particles in an isolated nucleus of CV-1 cell cultures. (B) Particles close to the plasma membrane and projecting into the intercellular space between two CV-1 cells potentially represent a cell-to-cell transmission event of SV40 particles. Such events were rarely detected. (C) Large numbers of SV40 viral particles attached to membranous structures.

Encouraged by the results from our negative staining EM, we next attempted to detect cell-associated virions in ultrathin (50 nm) resin sections of SV40 or MCVSyn-transfected cultures. 4 or 8 days after transfection with MCPyV or SV40 viral DNA, respectively, cells were trypsinized and absorbed in cellulose capillary tubes, followed by fixation, sectioning and EM analysis. In the SV40-transfected CV-1 cultures, approx. 20% of the cells were already lysed, and nearly all of the remaining cells showed massive virion production. Figure 9A shows an isolated but intact nucleus which is densely packed with viral particles, whereas Figure 9B shows the border of two live cells from which virion particles are shed and/or transmitted. We also frequently observed beads-on-a-string like structures that appear to represent viral particles associated with membranes; given the previously observed nuclear rim-like VP1 staining patterns [30], these may represent remnants of the nuclear envelope with fully assembled virons that were in the process of penetration and nuclear egress at the time of cell lysis (Figure 9C).

**Figure 10:**
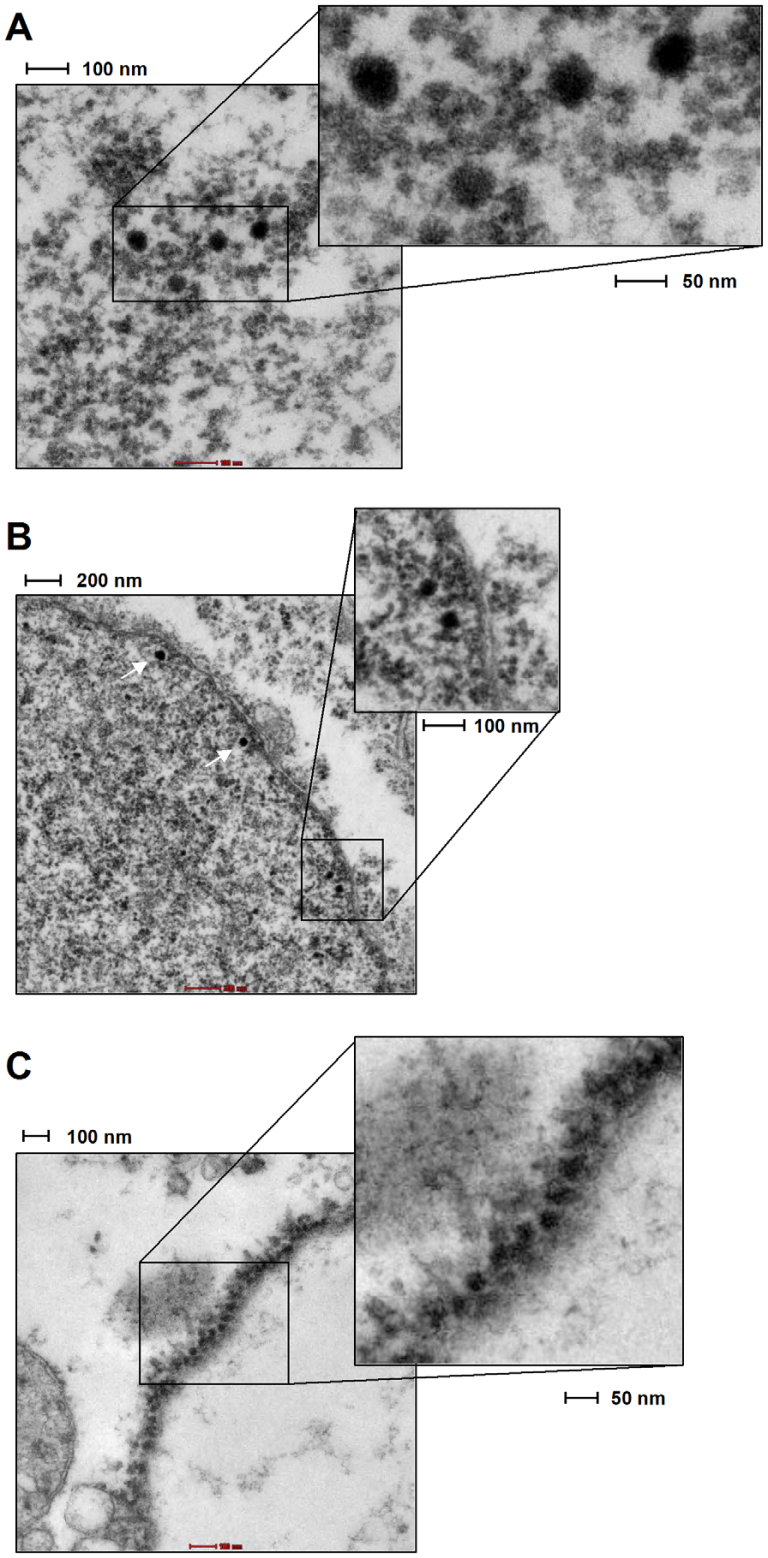
Electron micrographs of viral particles in MCVSyn-transfected PFSK-1 cells. Images were prepared from PFSK-1 cultures at 8 days post transfection with MCVSyn DNA. (A and B) ∼40 µm electron dense particles were observed in approximately 1 out of 50 cells with the particles localizing in the nucleus close to membrane structures (additional particles in B that are located outside of the enlarged inset are marked by arrows). (C) Membrane-attached MCPyV particles, reminiscent of the structures observed in SV40 infected cells as shown in Figure 9C.

In Figure 10, we present an EM analysis of PFSK-1 cells at 8 days post-transfection with MCVSyn DNA. In line with our previous observations, we were able to detect low-abundance electron dense particles that were in the size range expected for polyomavirus virions (Figure 10A). These particles were detected only in approximately 1 out of 50 cells, and preferentially localized to the nucleus and the nuclear periphery. We repeatedly observed particles in close proximity to nuclear membranes (Figure 10B), and also were able to detect particles attached to isolated, membrane-like structures (Figure 10C), reminiscent of our observations made in SV40-transfected CV-1 cells (compare Figures 9C and 10C). However, as already observed in our negative staining EM of gradient fractions, the MCPyV generally seemed to be slightly smaller and of less regular shape than SV40 cells. Based on our EM analysis, we estimate that MCPyV particles were at least 10,000 times less abundant than SV40 virions.

### No serial transmission of infectious particles

As indicated above, we did not observe a typical CPE in our MCVSyn-transfected cell cultures. Such an effect was also not apparent when we cultured the cells for extended periods of time (up to 48 days). Likewise, we did not observe evidence for horizontal spread (i.e., cell to cell transmission) in our cultures, as the total number of LT-Ag positive cells did not increase over time (data not shown). When we incubated fresh cells with supernatants transferred from MCVSyn-transfected H1299, PFSK1 or 293 cells at various time points post transfection, we were unable to detect early or late gene transcription, viral protein expression, or DNA replication. We also considered the possibility that due to the apparent absence of cell lysis, viral particles might not be efficiently released from transfected cells. However, even after multiple freeze/thaw cycles, we were unable to demonstrate serial transmission. It has been suggested that ganglioside GT1b serves as a receptor for MCPyV [33]. Although more recent data from Schowalter and colleagues argue against GT1b supporting attachment and/or entry for MCPyV, we nevertheless attempted to increase the permissiveness of the recipient cultures by addition of GT1b. Again, no serial transmission or viral spread was observed.

Taken together, these data suggest that the amount of viral particles is too low to achieve secondary infection, or that our target cells miss the appropriate entry receptor. Additionally, given the observed shape and size differences between MCPyV and SV40 particles, it appears possible that the virions themselves may be of low infectivity.

## Discussion

Here, we report a side-by-side analysis of SV40 and MCPyV replication, gene expression as well as virion production in a number of different human and animal cell lines. For our study, we generated a consensus MCPyV genome (termed MCVSyn) based on the consensus from thirteen partial as well as complete MCC-derived Merkel cell polyomavirus sequences. The fact that this consensus sequence is identical to three wild type/field strains isolated from human skin underlines the validity of our assumption that such a consensus should represent an authentic system to study the MCPyV lifecycle.

We observed that MCVSyn genomes readily undergo replication when transfected into a number of cell lines and/or primary cells. Efficient replication as well as detection of LT-antigen, however, was only seen in the three cell lines 293, H1299 and PFSK-1. In another established line, Saos-2, as well two primary cell cultures, HUVEC and NHDF, we failed to detect viral protein expression, but reproducibly observed weak and transient replication of transfected genomes (Figure S4).

It should be noted that the different cultures were naturally not equally susceptible to plasmid transfection (see Table 1), a fact that should be taken into account when comparing the absolute levels of replicated episomes as shown in Figures 4 and S4. For example, considering that transfection efficiencies in primary NHDF cultures were roughly 10fold lower than in 293, H1299 and PFSK-1 cells, it is likely that replication in primary dermal fibroblasts per se is no less efficient as in the three established cell lines (as discussed before, our failure to detect LT-Ag protein in these cells is likely to be a result simply of the limited sensitivity of our western blot detection).

Nonetheless, in some of the tested cell lines (e.g. HeLa or A549) no replication products were observed, despite of considerable transfection efficiencies, indicating that these lines are indeed less supportive of MCPyV replication, at least under conditions where the viral genome is delivered by transfection. Whether this is due to a failure to support early gene expression or origin-dependent replication itself remains to be established. It may also be possible that viral replication in some of these lines/cell types is negatively affected by differential expression of host cell factors, similar to the recently reported negative influence of the vacuolar sorting protein hVam6p on MCPyV replication [27].

At first glance, when comparing Figures 3 and 4 it seems counter-intuitive that H1299, 293 and PFSK-1 cells should accumulate increasing amounts of replication products at late time points post transfection, when at the same time LT-Ag protein levels decline over time. However, it should be pointed out that the bulk of LT-Ag expression at early time points is not produced from the replicated genomes, but rather from the input DNA that is sensitive to DpnI digestion and thus cleaved in the replication assays (see the smaller band/cleavage product in Figure 4). Thus, increased expression from accumulating replicated episomes is counterbalanced by a loss of expression from input DNA, a fact that is also reflected by the near-constant LT-Ag transcript levels as observed in Figure 6. It is likely that additional factors influence LT-Ag protein expression. For example, MCPyV has been shown to encode a microRNA (miRNA), mcv-mir-M1, that is located antisense to LT-Ag and that, based on in vitro luciferase assays, is able to negatively regulate LT-Ag expression [16]. It appears that usage of such miRNAs is a conserved feature of many polyomaviruses, a mechanism that presumably serves to tone down LT-Ag protein expression and thereby limit exposure of the infected cells to the immune system at late time points of infection (for a recent review on viral miRNAs, see [34]). While we verified that mcv-mir-M1 is expressed in our system (data not shown), we did not explicitly test to what extend it may contribute to the observed drop in LT-Ag protein levels. Further studies will be necessary to investigate the precise role of mcv-mir-M1 in the MCPyV lifecycle.

Regardless of the mechanism that leads to the decline of LT-Ag expression over time, we observed that stable co-expression of the early region of MCPyV resulted in significantly increased replication in 293 cells. As the early gene region is capable of expressing small as well as large T antigens, it is unclear whether this effect is due to the trans-complementation of LT-Ag loss, or reflects the indirect contribution of elevated ST-Ag levels to viral replication; a recent study suggests that the latter may be the case [27]. As noted before and shown in Figure 1, with the exception of LT-Ag truncating mutations in MCC-derived genomes, nucleotide insertions and deletions among MCPyV isolates are mainly observed in the non-coding control region (NCCR) of the virus, including the replication core. Some of these mutations have been shown to positively affect viral replication, such as the duplication of a GGGNGGRR motif present in the MCPyV strains TKS and 16b [7], [35]. We compared MCVSyn to another field isolate, R17a, which differs from the consensus sequence only in the deletion of a single nucleotide in the core origin; the same deletion is also present in isolate 09b. Interestingly, the R17a isolate replicated with a markedly lower efficiency than the MCVSyn genome (Figure 5), indicating that replication efficiency may indeed differ significantly among field isolates of Merkel cell polyomavirus.

Although we did not observe cytopathic effects/cell lysis or serial transmission, using electron microscopy we were nevertheless able to detect viral particles with a diameter of approximately 40 nm in MCVSyn transfected cells, both by negative staining of gradient fractions as well as in intact cells. In accord with the results from our replication and expression analyses, virions were much less abundant than in SV40-transfected CV-1 cells. For gradient fractions as well as cell-bound virions, we found MCPyV particles to be slightly smaller (40 nm) and of a less regular shape than SV40 particles; while both could be intrinsic properties of MCPyV virions, this observation suggested to us that the particles produced in this system might not be completely mature, which may in part explain the lack of serial transmission.

Polyomavirus replication, especially in the SV40 system, is well studied, however far less is known about virus assembly and the particular signals that trigger and control this process. We observe efficient expression of VP1 protein in the nucleus of SV40 DNA transfected CV-1 cells, and at 72 h post transfection find the protein to localize almost exclusively at the nuclear rim. Similar data are known from JC Virus where VP1 localization is also reported at the nuclear rim in granular structures most likely resembling sites of virus assembly [32]. Although we were unable to directly compare VP1 protein expression levels by western blotting, in immunofluorescence analyses of MCVSyn-transfected cells we observed VP1 staining as early as 4d post transfection. Interestingly, although strictly nuclear, MCPyV VP1 protein localizes to more perinuclear regions in the nucleus, and not strictly at the nuclear envelope. Similar localization pattern of VP1 proteins has been observed for JC-reporter mutants that overexpress the complete late gene region but lack VP3 protein [32]. It has been speculated that, due to a lack of the conserved Met-Ala-Leu motif that has been identified in all polyomavirus VP3 proteins so far, a functional VP3 protein might not be present in MCPyV [18]. For SV40, perinuclear staining of VP1 protein has been reported in the absence of the agnoprotein, which is also important for viral release [36], [37]. As no agnoprotein has been annotated for the MCPyV sequence [2], [13], both the potential lack of a functional VP3 protein as well as an agnoprotein may impede proper localization of the VP1 protein, at least in the cell types and replication system tested here. Lastly, we note that proper timing of viral gene expression may be a factor that could contribute to the efficiency of virion assembly. Especially since polyomavirus particles package chromatinized genomes, it is likely that fine tuning of the viral gene expression program may differ from the transfection of naked DNA. Ultimately, the answer to this question will have to await the availability of a system that is fully permissive for viral infection.

While our manuscript was in preparation, two studies investigating MCPyV replication and transmission *in vitro* were reported [27], [38]. Feng and colleagues used an approach very similar to ours, i.e. a consensus viral genome that is identical in sequence to MCVSyn, to study viral replication and transmission in 293 cells [27]. In agreement with our data, Feng et al. observed DNA replication and particle production, but no serial transmission. Schowalter et al. performed gradient-purification and enrichment of virion-containing fractions from freeze/thawed 293-4T cells (293T cells that stably express an extra copy of SV40 LT-Ag, as well as the small and large T Antigens of Merkel cell polyomavirus) transfected with R17a genomes [27], [38]. Using this highly optimized system, the authors were able to detect an increase of viral DNA relative to input DNA when the enriched fractions were transferred to fresh cultures of 293-4T cells, suggesting replication of genomes delivered by infectious virions. However, in agreement with our data and those of Feng et al., the observed two-fold enrichment of genomic DNA over input DNA suggests a low efficiency of transmission. While we did not attempt to detect viral particles produced by R17a-genomes, we did ensure that LT-Ag and VP1 expression and localization do not differ fundamentally between MCVSyn- and R17a-transfected H1299 and PFSK-1 cells (Figure S6). Given the higher DNA replication efficiency observed in our replication assays (Figure 5), it will therefore be interesting to test the MCVSyn genome in highly sensitive infection systems such as the one used by Schowalter et al. [27], [38].

In summary, the semi permissive *in vitro* replication system reported here provides an excellent starting point for further analyses of the MCPyV lifecycle. The availability of genetically tractable genomic clones such as MCVSyn will greatly facilitate the study of the role of viral as well as cellular factors during Merkel cell polyomavirus replication, virion assembly and egress.

## Materials and Methods

### Cell lines and tissue culture

Human cell lines 293 cells, 293T cells [39], Bea2b cells (ATCC, CRL-9609), H1299 cells [40], Saos-2 cells [41], A549 [42], HaCaT cells [43], HeLa cells (ATCC, CCL-2) and mouse N2A cells (ATCC, CCL-131), were grown as monolayer cultures in Dulbecco's modified Eagle's medium (DMEM) supplemented with 10% fetal calf serum (FCS) and 5% penicillin/streptomycin in a 5% CO_2_ atmosphere at 37°C. Human PFSK-1 cells (ATCC, CRL-2060), the human neuroblastoma cell lines LAN-1 [44], IMR-32 [45] and SH-SY5Y [46] and African green monkey CV1 (ATCC, CCL-70), Vero cells (ATCC, CCL-81), and COS7 cells (ATCC, CRL-1651) were grown in RPMI medium supplemented with 10% FCS and 5% penicillin/streptomycin. HUVEC cells were maintained in endothelial cell basal medium (Cambrex, Bioscience) supplemented with 10% FCS and EGM Single Quots (Cambrex Bioscience). Only cells maintained for 5 to 10 passages were used in experiments.

Normal human dermal fibroblasts (NHDF) were purchased from Lonza, cultured in fibroblast ready to use growth medium supplemented with FCS and insulin (Lonza). Primary human keratinocytes (Lonza) were maintained in keratinocyte basal media supplemented with the corresponding BulletKit. 293 cells stably expressing MCPyV LT- Ag were generated by transfection of cells with linearized pCMV2a-MCPyV LT [47] followed by bulk selection using G418.

### Plasmids and transfection methods

To generate the synthetic clone MCVSyn, we first generated a MCPyV consensus sequence by aligning 13 partial and complete MCC-derived MCPyV sequences (see below for accession numbers) deposited in the NCBI database as of June 2008. The CLUSTAL W algorithm was used to produce the alignments, and sequence conflicts were resolved by majority vote among the individual isolates. The resulting MCVSyn consensus sequence was assembled synthetically in two fragments inserted into the cloning vector pMK (GeneART, Regensburg). pMK-MCVSyn-A contains nucleotides 1549-4540, whereas pMK-MCVSyn-B contains nucleotides 4528-1580 of the MCVSyn genome. In both constructs, the inserts are flanked by PacI and/or SacI sites (see Figure S1). A complete synthetic MCVSyn genome in which the early region is disrupted by the pMK vector backbone sequence, pMK-MCVSyn, was constructed by releasing a 2452 bp from pMK-MCVSyn-B by PacI digestion, and inserting it into the PacI-linearized pMK-MCVSyn-A vector. To produce circularized MCVSyn DNA for transfection, the viral genome was released from pMK-MCVSyn by SacI digestion (Figure S1). Subsequently, intramolecular ligation of viral DNA was performed using T4 DNA Ligase overnight, followed by column purification of recircularized DNA as described [48]. The sequences of pMCVSyn and MCVSyn have been deposited in GenBank (accession numbers JN707598 and JN707599, respectively).

Full-length genomes of SV40 and MCPyV isolate R17a (GenBank accession HM011555.1) were produced via EcoRI- or BamHI-mediated excision from plasmids pMCV-R17a [7] or pSV40 [49], respectively, followed by intramolecular religation of the viral DNA using T4 DNA Ligase and subsequent column purification of the circularized DNA [48]. Plasmid pMCV-R17a was created by Schowalter et al. [7] and was obtained from Addgene (www.addgene.org, plasmid #24729). pSV40 [49] was generously provided by Wolfram Deppert, Heinrich-Pette Institute, Hamburg. pCMV2-MCPyV LT encodes LT- as well as ST-Ag and of MCPyV and was generated by PCR amplification of the early gene region from re-circularized MCVSyn DNA, using primers MCPyV_EcoRV_F and XhoI MCPyV_XhoI_R (see Table S2 for primer sequences) and the taqExpand PCR amplification system (Roche Diagnostics). Plasmid pwM [7] expresses a codon optimized MCPyV VP1 ORF (GenBank accession FJ548568) and was obtained from Addgene (plasmid #22515).

HUVEC cells were transfected using the Neon™ nucleofactor system (Invitrogen), and HaCaT as well as primary human keratinocytes were transfected by AMAXA Nucleofector II technology, following the manufacturer's instructions. All other cell lines were transfected using the FuGENE HP transfection reagent (Roche diagnostics) according to the manufacturer's instructions. Saos-2 cells were transfected in the presence of ExGen500 (Fermentas Inc.).

### Sequence analysis

Multiple sequence alignments and generation of phylogenetic trees were performed using the ClustalW2 server at EMBL-EBI (www.ebi.ac.uk/Tools/msa/clustalw2). The accession numbers for all sequences used in the analyses shown in Figs. 1 and S1 are given in Table S3. The following MCC-derived MCPyV sequences were used to build the MCVSyn consensus genome (see Table S3 for accession numbers): MCC206, MCC322, MCC339, MCC344, MCC345, MCC347, MCC348, MCC349, MCC350, MCC352, MCC366, MCC85, MKL-1.

### Viral DNA Replication assay

1×10^5^ cells were transfected with 100 ng MCVSyn DNA and 250 ng carrier DNA (pUC18) or equivalent amounts of carrier DNA only (mock control). At 2, 4, 8 and 12d post transfection, low molecular weight DNA was purified by HIRT extraction as described before [50]. 1 µg of the isolated DNA was digested with DpnI and EcoRI for 30 min at 37°C (FASTdigest, Fermentas) and separated on a 0.8% DNA Agarose gel. DNA was transferred to Hybond N+ membrane (GE Healthcare) and analyzed by Southern blot analysis using a ^32^P dCTP labelled PCR amplified LT-fragment (rediPrime, GE Healthcare) resuspended in ULTRAhyb Solution (Ambion) as a probe. THe primers used to generate the probes were MCPyV-LT-s and MCPyV-LT-as (see Table S2). Blots were hybridized for 16 h at 42°C and subsequently washed 2×15 min 2%SSC, 0.1%SDS followed by 1×15 min 0.2×SSC, 0.1%SDS at 42°C. Blots were exposed for 24 h and subsequently scanned using Fuji phosphoimager FLA7000 and MultiGauge software.

### Antibodies and Western Blotting

Cells were resuspended in lysis buffer (50 mM Tris pH 8.0, 150 mM NaCl, 1% NP40, 0.5% Na-Deoxycholat, 5 mM EDTA, 0.1% SDS, proteinase inhibitor cocktail, Roche). 25 µg total protein per lane was separated by 10% SDS PAGE and blotted on a PVDF membrane. Primary antibodies specific for polyomavirus proteins used in this study and their suppliers were: mouse monoclonal (mAb) MCPyV LT-Ag antibody Cm2B4 [14] (Santa Cruz), mouse monoclonal SV40 LT antibody Pab419 [51] (Santa Cruz) and MCPyV VP1 rabbit polyclonal antiserum [18] generously provided by Chris Buck, NIH. An actin mAb (Chemicon Cat.No. 1501) was used to ensure equal protein amounts loaded. Secondary antibodies conjugated to horseradish peroxidase (HRP) for the detection of proteins by immunoblotting were anti-mouse IgG (GE Healthcare) and anti-rabbit IgG (Santa Cruz).

### Immunofluorescence staining and confocal microscopy

For indirect immunofluorescence, cells were grown on glass cover slips coated with 0.2% gelatine (Sigma). Cells were fixed in 4% paraformaldehyde in PBS (15 min) and permeabilized in PBS, 3% Triton X-100 for 30 min. After 1 h blocking in PBS containing 1% Triton X-100, 0.5% Tween, 3% BSA (Albumin fraction) buffer, coverslips were incubated for 3 h with the primary antibodies diluted in blocking buffer, washed in PBS, followed by incubation with the corresponding fluorophor conjugated secondary antibodies (Dianova, Hamburg, Germany). Coverslips were mounted in vectashield medium containing DAPI (Vectashield) and digital images were acquired with a confocal laser-scanning microscope (Leica DM IRE2 with a Leica TCS SP2 AOBS confocal point scanner) equipped with an oil-immersion plan Apo 63× NA 1.4 objective.

### Determination of LT and VP1 transcript by quantitiative PCR

Total RNA of MCVSyn transfected or MOCK transfected cells was isolated using the RNeasy extraction kit (Qiagen, CatNo. 74104) according to manufacturer's instructions. DNaseI digest was performed following the on-column protocol provided by Qiagen. 500 ng total RNA was reverse transcribed with a random hexamer primer and the SuperScriptTM Reverse Transcriptase (Invitrogen, CatNo. 18064-014). cDNA levels were determined using a Rotorgene Q 5plex instrument (Qiagen) and Rotorgene 1.7 software. Reactions were performed in microtubes containing 5 µl 2× SyBr Green mastermix (Fermentas), 3.8 µl H_2_O, 0.1 µl primer and 1 µl (20 µl) cDNA. Primer sequences, annealing temperatures and PCR efficiency are listed in Table S2. PCR efficiency of each primer set was determined by standard curves of serial 10fold dilutions of plasmid DNA (LT: pCMV-MCPyV LT; VP1: pCMV-MCPyV VP1. Ct values (determined by the Rotorgene Software version 1.7) were plotted against the log10 value of template concentration, with the slope (M) determining the reaction efficiency according to the formula (10-1/M)-1 = 1. Relative expression levels were calculated by incorporating the PCR efficiency according to the Rotorgene Software version 1.7. Relative mRNA levels were normalized to GAPDH (see Table S2 for the primer sequence).

### Transmission electron microscopy

For ultrastructural analysis of virus assembly processes cellulose capillary tubes of 200 µm diameter [52] were filled with cells 4–6d past transfection with viral DNA for 4–6d by capillary attraction and mechanically sealed at both ends using the blunt side of a scalpel. Chemical fixation of the cells was accomplished by incubating the highly porous tubes (molecular weight cut-off limit 10 kDa) with 2.5% glutaraldehyde (GA) in PBS for 30 minutes at room temperature. Subsequently, the cells were washed with PBS, postfixed for 30 minutes with 1% OsO_4_ in PBS, washed with ddH_2_O, and stained with 1% uranyl acetate in water. The samples were gradually dehydrated with ethanol and embedded in Epon resin (Carl Roth, Germany) for sectioning. Ultrathin 50 nm sections were prepared using Ultracut Microtome (Leica Microsystems, Germany). The sections were counterstained with 2% uranyl acetate and lead citrate.

For negative staining, 15 µl of the different purified virus fractions were applied on Parafilm and covered with a glow discharged carbon-coated Formvar grid (mesh size, 200) for 15 min. After adsorption, grids were fixed with glutaraldehyde 2.5% for 2 min, were washed three times with aqua dest. and stained with 2% uranyl actetate for 5 min. Excess stain was soaked off by touching the grid to a filter paper. After staining, the grids were air dried. Electron micrographs were obtained with a FEI Eagle 4 k HS CCD camera attached to a FEI Technai G 20 Twin Transmission Electron Microscope (FEI, Eindhoven, The Netherlands) at 80 kv.

### Purification of virion particles by ultracentrifugation using OptiPrep™

4d p.t. cells transfected with viral DNA were lysed (PBS supplemented with 9.5 mM MgCl2, 0.5% Triton X-100, 1% pen-strep-mix (Gibco), 0.1% RNaseA) and incubated over night at 37°C to support particle formation. After the lysate was clarified by centrifugation (2×10 min at 10,000*g), 120 µl of lysate was loaded onto an OptiPrep™ (iodixanol) gradient (27-33-39%) as described [53] and centrifugated for 4 h at 52,000 rpm using an SW60Ti Rotor. Tubes were punctured and 250 µl fractions were collected. Fractions were analyzed for the presence of SV40 virions using Western Blotting, applying a VP1 antibody (α-SV40 VP1 polyclonal serum kindly provided by Wolfgang Deppert, Heinrich-Pette Institute, Hamburg), or by testing the fractions for infection of CV-1 cells (successful infection was monitored by IFA or CPE). All fractions were micrococcal nuclease treated by incubating 20 µl of the fraction with 40 U micrococcal nuclease for 1 h at 37°C, followed by stopping of the reaction with EGTA. Undigested/virion incorporated DNA was extracted by Phenol/Chloroform extraction, followed by Chloroform/Isoamylalcohol extraction and Ethanol precipitation. DNA was resuspended in 20 µl water and 1/10^th^ was analyzed by realtime PCR using the primers listed in Table S2. In the case of Merkel cell polyomavirus, fractions were analyzed for the presence of MCPyV particles by negative EM staining.

### SV40 virus production

CV1 cells were seeded at 40–50% confluence one day prior infection in 10 cm dish. Medium was replaced with 1.5 ml medium containing SV40 cell lysate (passed through a 0.45 µm filter) MOI 0.1. After 2 h incubation at 37°C in an incubator, 8 ml fresh culture medium was added. Cells were harvested once a complete CPE was visible. Cells and floating cell debris were transferred to a T75 culture flask and three cycles of freeze and thawing of the cells was performed. Cellular debris was removed by centrifugation (200RCF 5 min) and serial dilutions were generated to determine SV40 Titer. Titering was performed by infecting CV-1 cells and subsequent immunofluorescence analysis for the expression of LT-Ag 36 h p.i.

### Serial transmission of MCPyV using lysates from MCPyV transfected cells

Virus lysates were generated by resuspending 5×10^5^ MCPyV-transfected cells at 4d p.t. in 500 µl culture medium, performing three freeze/thaw cycles followed by removal of cellular debris through low speed centrifugation or passing through a 0.45 µm filter. Prior to the day of infection, 5×10^4^ cells of 293, PFSK-1 and H1299 were seeded in 12well plates. On the day of infection culture medium was replaced with 500 µl medium containing 500 µl, 250 µl, 100 µl and 50 µl of virus lysates from 293, PFSK-1 or H1299 cells, in the presence or absence of polybrene at a final concentration of 8 µg/ml. GT1b (Sigma) was added (3.2 µM final concentration) 18 h prior infection. Infected cultures were monitored for MCPyV infection by performing immunofluorescence for viral gene products and viral DNA replication assays.

## Supporting Information

Figure S1 **Generation of the consensus MCVSyn genome.** The consensus MCPyV genome was synthesized as two separate fragments which were inserted into the cloning vector pMK: pMK-MCVSyn-B contains nucleotides 4528-1580 flanked by PacI restriction sites; whereas pMK-MCVSyn-A contains nucleotides 1549-4540 (all nucleotide positions are given relative to GenBank entry HM011549). The MCVSyn-B PacI insert was cloned into the PacI-linearized pMK-MCVSyn-A to create pMK-MCVSyn, a construct which carries the complete MCPyV genome with the early gene region being disrupted by the vector backbone- A 13 bp duplication that contains a SacI site is present on each side of the viral genome and can be used to release the MCVSyn genome, followed by intramolecular recircularization using T4 DNA Ligase.
(TIF)

Figure S2 **Phylogenetic tree of full length MCPyV genomes including MCVSyn.** Phylogenetic analysis of MCVSyn and all full length MCPyV sequences deposited in the NCBI Database as of August 2011 (see Table S3 for accession numbers).
(TIF)

Figure S3 **Cytopathic effect (CPE) of CV-1 cells transfected with SV40 viral DNA.** CV-1 transfected with SV40 DNA start to show irregular round shaped cells which are enlarged and contain dense bodies at 4 days post transfection. Cells start to detach at day 6, and cultures are completely lysed by day 11.
(TIF)

Figure S4 **MCVSyn replication assays in HUVEC, NHDF and Saos-2 cells.** Low molecular weight DNA was isolated by HIRT extraction, 1.5 µg (HUVEC), 1 µg (NHDF) or 2 µg (Saos-2) DNA was DpnI and EcoRI digested, separated on an agarose gel and transferred to Hybond N+ membrane. DNA was probed with a radioactively labelled LT-Ag PCR fragment. The Blot was exposed for 24 h and scanned using Fuji phosphoimager FLA7000 and MultiGauge software.
(TIF)

Figure S5 VP1 and LT double staining in SV40 transfected CV-1 cells (A) and MCVSyn transfected H1299 cells (B). Cells transfected with intramolecular religated viral DNA were fixed at 4 day post transfection. VP1 was visualized with specific rabbit polyclonal VP1 antisera and anti-rabbit FITC staining while LT-Ag was visualized using specific monoclonal Ab and subsequent anti-mouse TRITC staining. DNA was stained by DAPI.
(TIF)

Figure S6 **Detection of LT-Ag and VP1 in an replication assay using religated R17a viral DNA in H1299 and PFSK-1 cells.** Cells were transfected with 100 ng religated DNA; 4d.p.t. cells were examined for LT-Ag expression and VP1 protein expression by immunofluorescence double staining. VP1 protein was visualized using polyclonal rabbit anti-VP1 serum and anti-rabbit FITC, whereas LT-Ag was visualized using monoclonal mouse Cm2B4 antibody and anti-mouse TRITC. Z-stack pictures were taken at 63× magnification, 2× zoom using confocal microscopy. Each picture represents an image from a single image from a Z-stack. VP1 staining was observed throughout the nucleoplasm, with some protein localizing to subnuclear speckles while LT-Ag staining was observed as granular staining throughout the nucleus.
(TIF)

Table S1 Sequence variation between MCVSyn and MCPyV isolates R17a, R17b and R30a.
(PDF)

Table S2 Summary of primer sequences and PCR conditions.
(PDF)

Table S3 Accession numbers.
(PDF)
