# Supplementary figures and images for: Replication, Gene Expression and Particle Production by a Consensus Merkel Cell Polyomavirus (MCPyV) Genome
Source: PLoS One. 2011 Dec 27;6(12):e29112. doi: 10.1371/journal.pone.0029112 (PMC3246459; doi:10.1371/journal.pone.0029112)

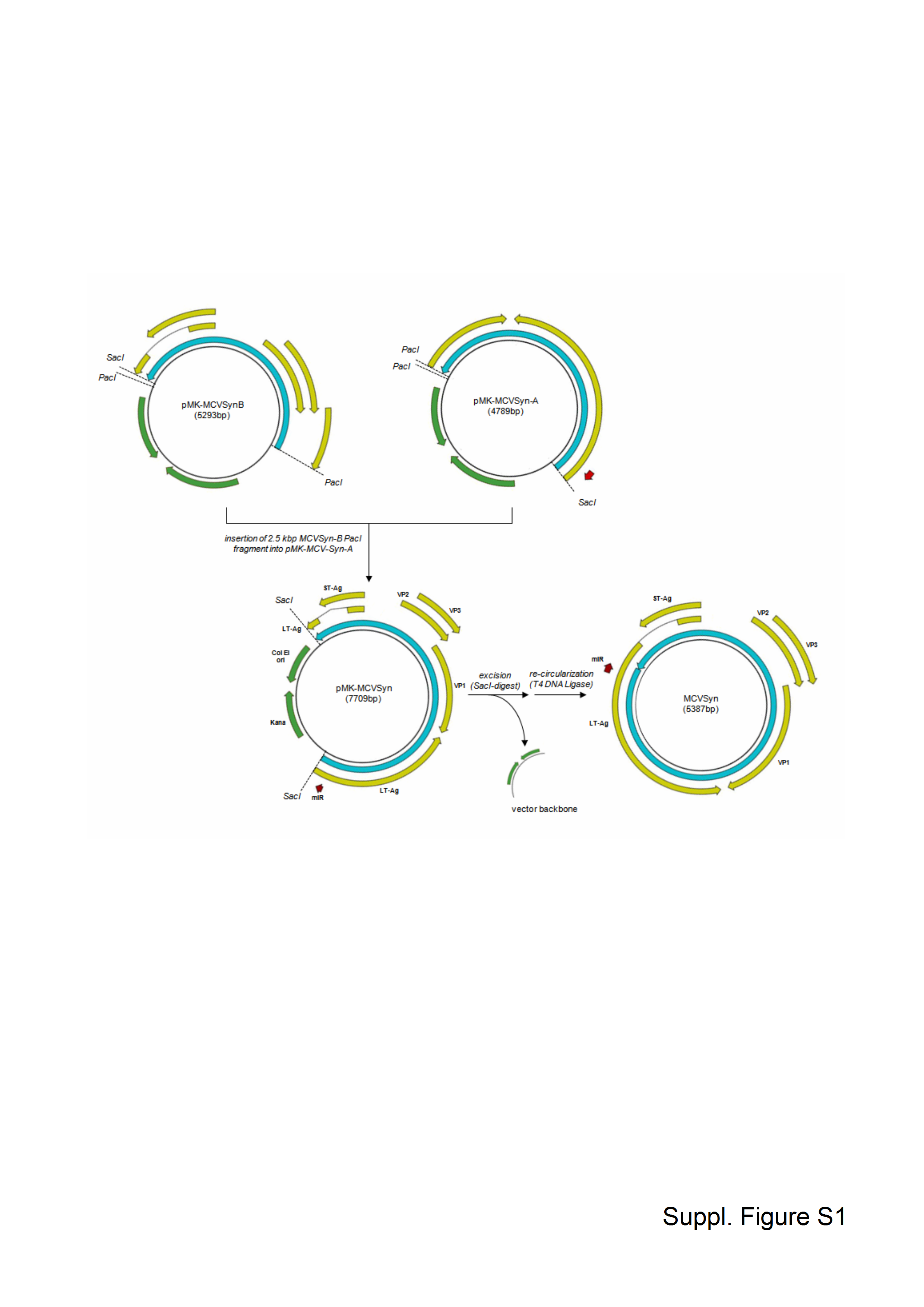

Supplement: Figure S1 — Generation of the consensus MCVSyn genome. The consensus MCPyV genome was synthesized as two separate fragments which were inserted into the cloning vector pMK: pMK-MCVSyn-B contains nucleotides 4528-1580 flanked by PacI restriction sites; whereas pMK-MCVSyn-A contains nucleotides 1549-4540 (all nucleotide positions are given relative to GenBank entry HM011549). The MCVSyn-B PacI insert was cloned into the PacI-linearized pMK-MCVSyn-A to create pMK-MCVSyn, a construct which carries the complete MCPyV genome with the early gene region being disrupted by the vector backbone- A 13 bp duplication that contains a SacI site is present on each side of the viral genome and can be used to release the MCVSyn genome, followed by intramolecular recircularization using T4 DNA Ligase. (TIF) [file pone.0029112.s001.tif]

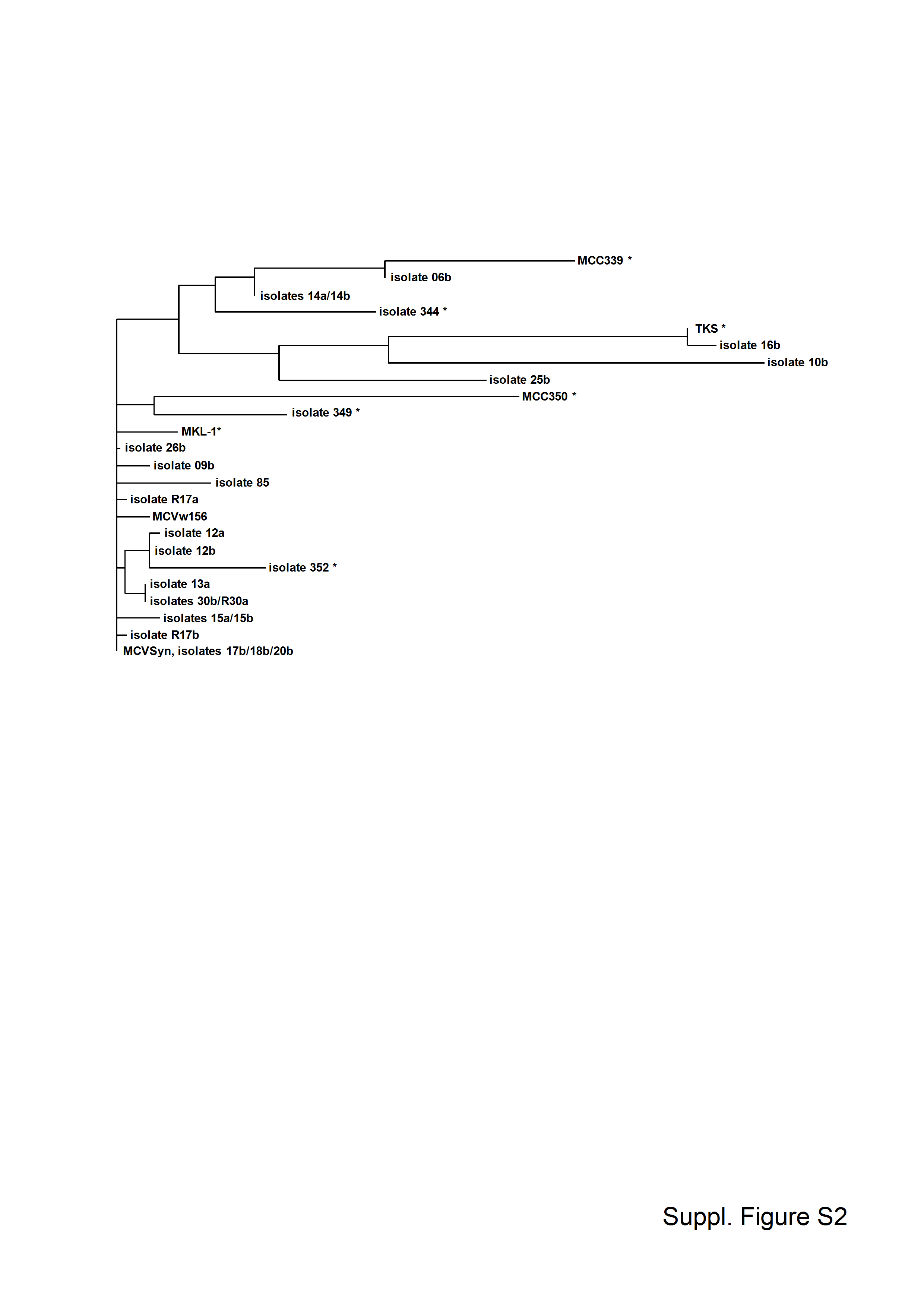

Supplement: Figure S2 — Phylogenetic tree of full length MCPyV genomes including MCVSyn. Phylogenetic analysis of MCVSyn and all full length MCPyV sequences deposited in the NCBI Database as of August 2011 (see Table S3 for accession numbers). (TIF) [file pone.0029112.s002.tif]

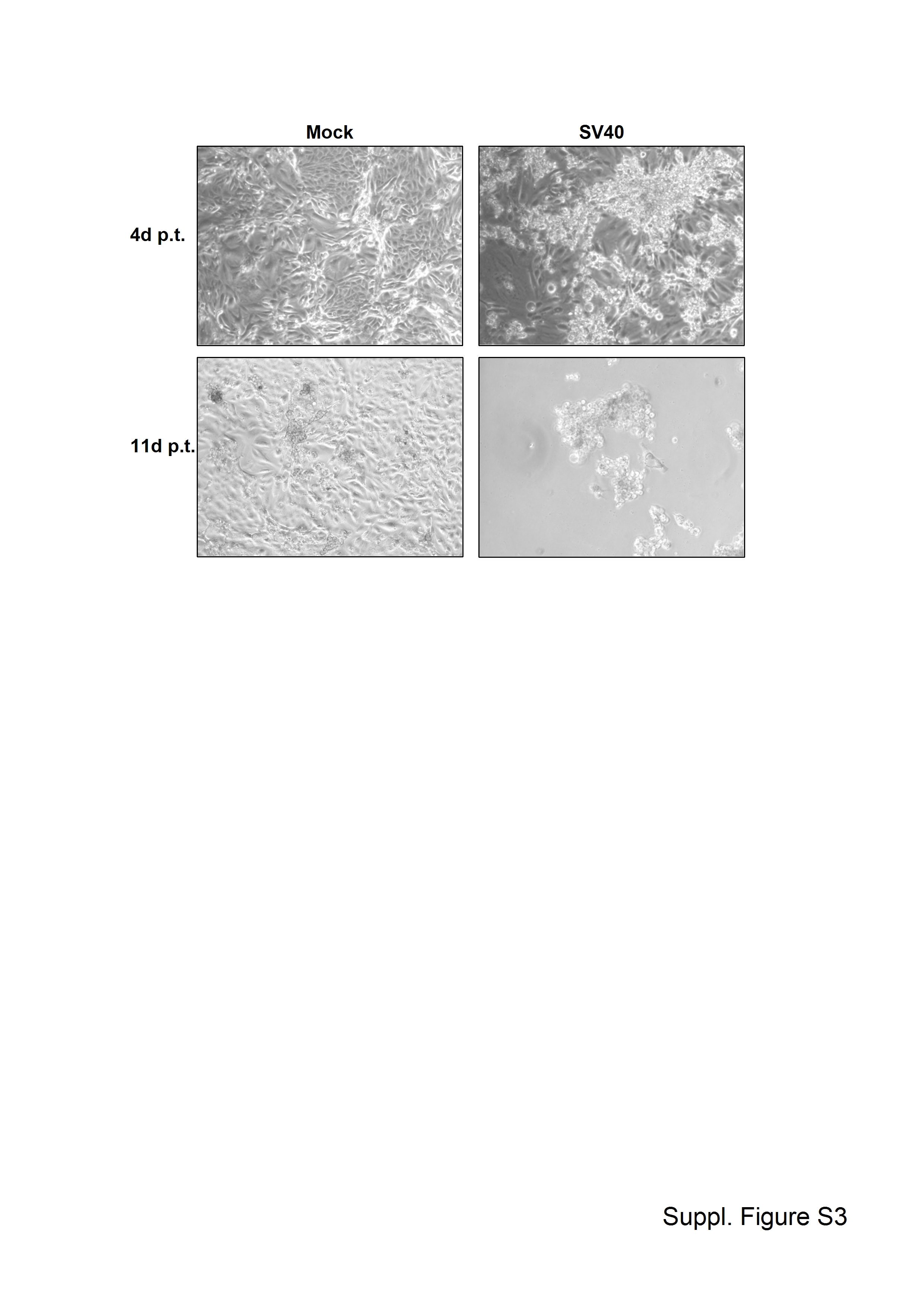

Supplement: Figure S3 — Cytopathic effect (CPE) of CV-1 cells transfected with SV40 viral DNA. CV-1 transfected with SV40 DNA start to show irregular round shaped cells which are enlarged and contain dense bodies at 4 days post transfection. Cells start to detach at day 6, and cultures are completely lysed by day 11. (TIF) [file pone.0029112.s003.tif]

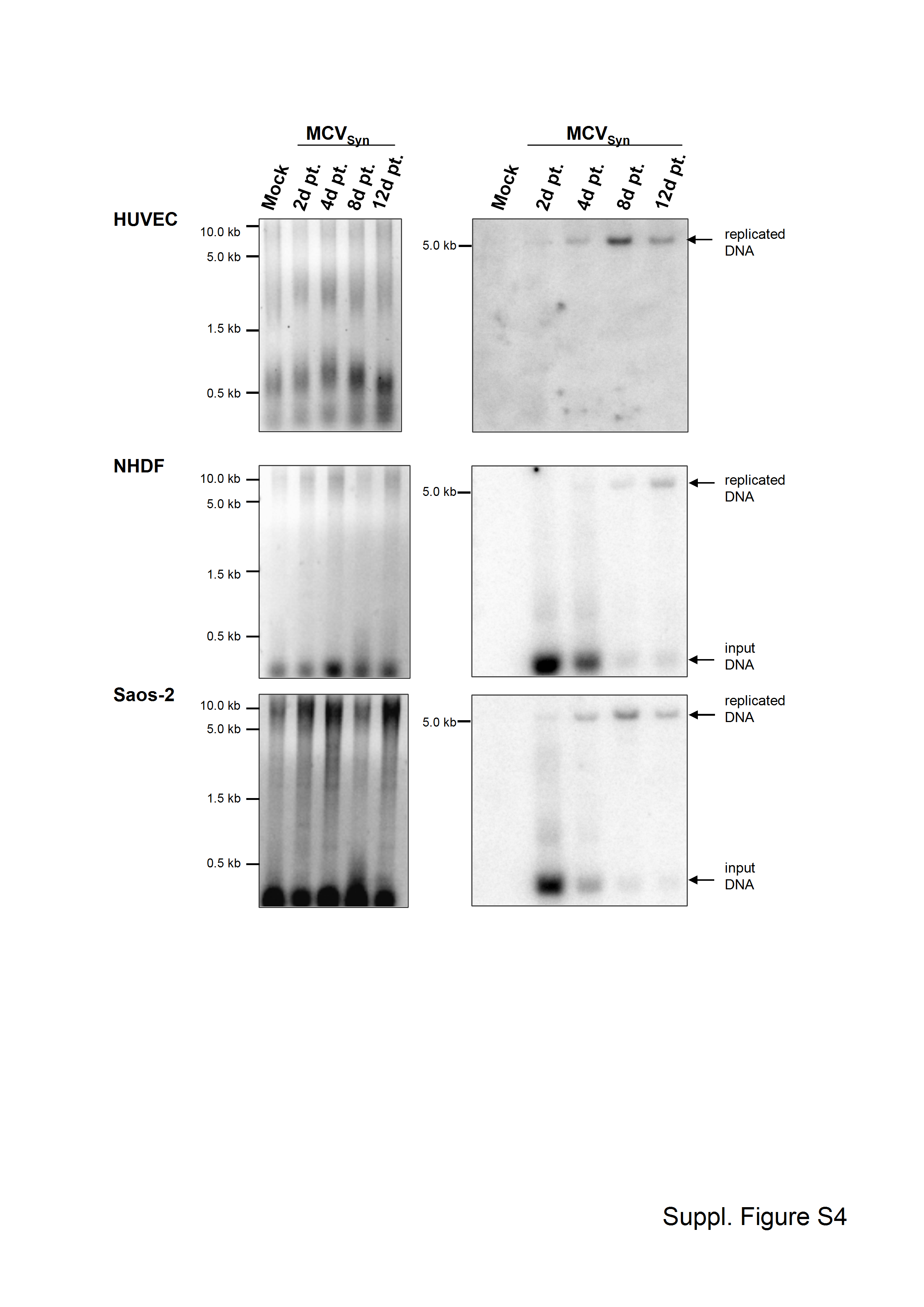

Supplement: Figure S4 — MCVSyn replication assays in HUVEC, NHDF and Saos-2 cells. Low molecular weight DNA was isolated by HIRT extraction, 1.5 µg (HUVEC), 1 µg (NHDF) or 2 µg (Saos-2) DNA was DpnI and EcoRI digested, separated on an agarose gel and transferred to Hybond N+ membrane. DNA was probed with a radioactively labelled LT-Ag PCR fragment. The Blot was exposed for 24 h and scanned using Fuji phosphoimager FLA7000 and MultiGauge software. (TIF) [file pone.0029112.s004.tif]

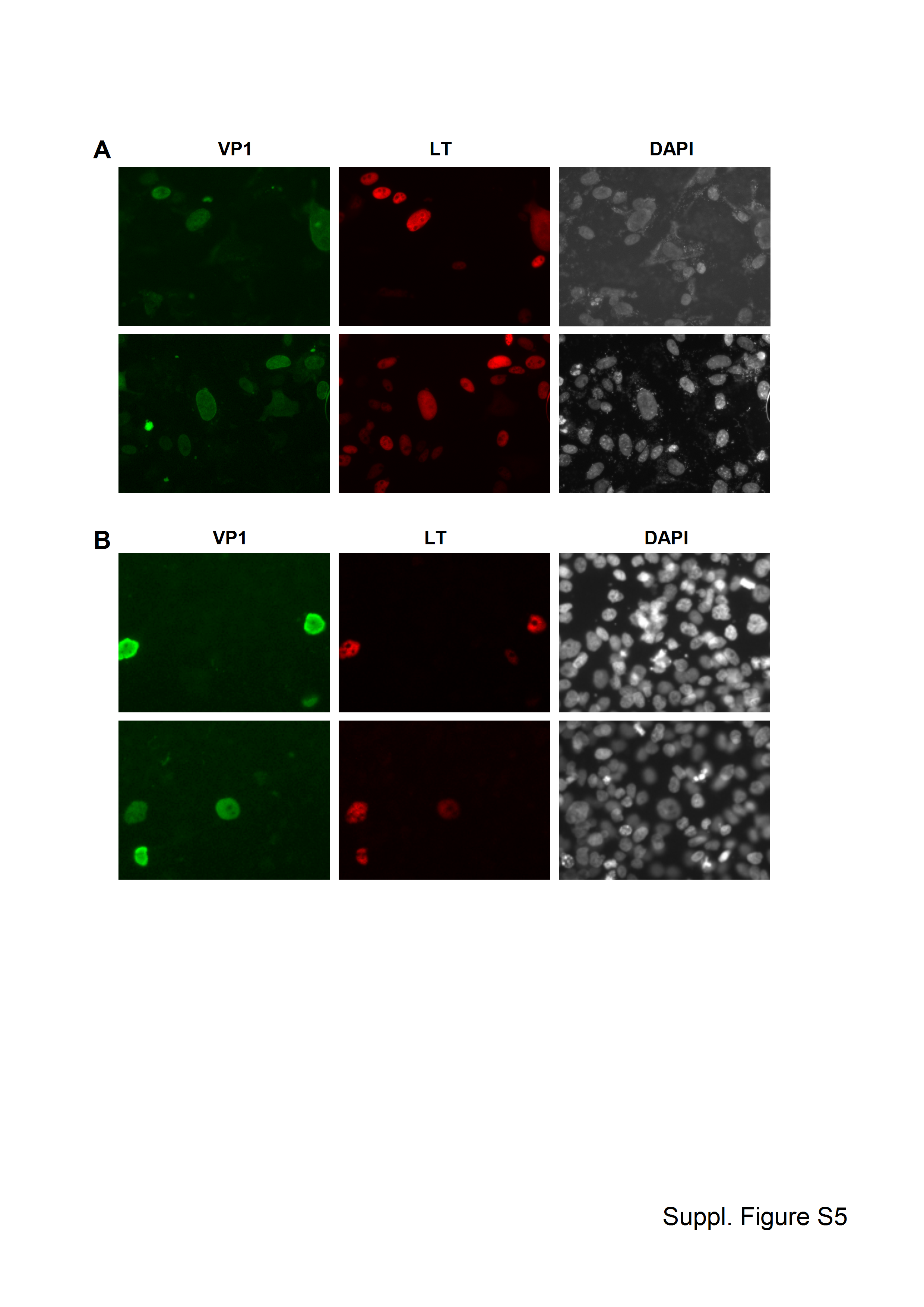

Supplement: Figure S5 — VP1 and LT double staining in SV40 transfected CV-1 cells (A) and MCVSyn transfected H1299 cells (B). Cells transfected with intramolecular religated viral DNA were fixed at 4 day post transfection. VP1 was visualized with specific rabbit polyclonal VP1 antisera and anti-rabbit FITC staining while LT-Ag was visualized using specific monoclonal Ab and subsequent anti-mouse TRITC staining. DNA was stained by DAPI. (TIF) [file pone.0029112.s005.tif]

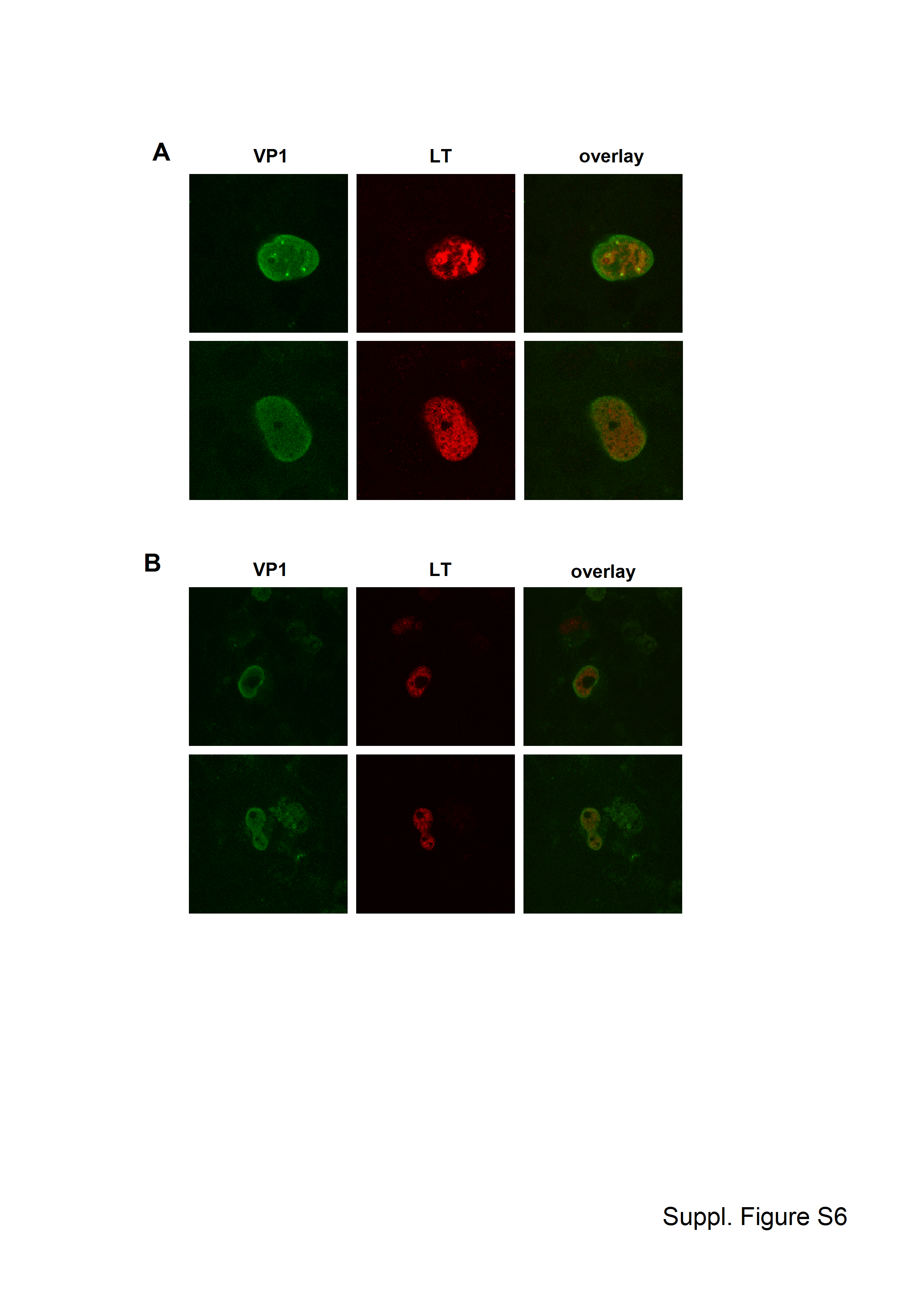

Supplement: Figure S6 — Detection of LT-Ag and VP1 in an replication assay using religated R17a viral DNA in H1299 and PFSK-1 cells. Cells were transfected with 100 ng religated DNA; 4d.p.t. cells were examined for LT-Ag expression and VP1 protein expression by immunofluorescence double staining. VP1 protein was visualized using polyclonal rabbit anti-VP1 serum and anti-rabbit FITC, whereas LT-Ag was visualized using monoclonal mouse Cm2B4 antibody and anti-mouse TRITC. Z-stack pictures were taken at 63× magnification, 2× zoom using confocal microscopy. Each picture represents an image from a single image from a Z-stack. VP1 staining was observed throughout the nucleoplasm, with some protein localizing to subnuclear speckles while LT-Ag staining was observed as granular staining throughout the nucleus. (TIF) [file pone.0029112.s006.tif]
