# Supplementary material for: Replication, Gene Expression and Particle Production by a Consensus Merkel Cell Polyomavirus (MCPyV) Genome
Source: PLoS One. 2011 Dec 27;6(12):e29112. doi: 10.1371/journal.pone.0029112 (PMC3246459; doi:10.1371/journal.pone.0029112)
Supplement: Table S1 — Sequence variation between MCVSyn and MCPyV isolates R17a, R17b and R30a. (PDF) [file pone.0029112.s007.pdf]

Supplementary Table S1: Sequence variation between MCVSyn and MCPyV isolates R17a, R17b and R30a

isolate	accession	nuc. sequence (MCVSyn/isolate)	nuc. position[a]	gene/region	amino acid (MCVSyn/isolate)	amino acid position[a]
R30a	HM011557.1	T/ C	1693	LT-Ag	Ile/ Ile	589
R30a	HM011557.1	T/C	2596	LT-Ag	Glu/Glu	786
R30a	HM011557.1	A/G	3190	VP1	Leu/Leu	180
R17a	HM011555.1	A/-	168	NCCR[b]	n.a.	n.a.
R17b	HM011556.1	G/A	1203	VP1	Arg/Arg	16

[a] all positions are based on GenBank entry HM011549.1
[b] NCCR: non-coding control region
