# Supplementary material for: Replication, Gene Expression and Particle Production by a Consensus Merkel Cell Polyomavirus (MCPyV) Genome
Source: PLoS One. 2011 Dec 27;6(12):e29112. doi: 10.1371/journal.pone.0029112 (PMC3246459; doi:10.1371/journal.pone.0029112)
Supplement: Table S2 — Summary of primer sequences and PCR conditions. (PDF) [file pone.0029112.s008.pdf]

Supplementary Table S2: Summary of primer sequences and PCR conditions

	PCR primer sequences 5'-3'	Tm	PCR efficiency[a]	References
GAPDH-fw	GAAGGTGAAGGTCGGAGTC	60°C	0.94	Urisman et al., 2004
GAPDH-rv	GAAGATGGTGATGGGATTTC	60°C	0.94	Urisman et al., 2004
SV40-VP1-fw	TGTTGACATTTGTGGGCTGT	60°C	1.15	This study
SV40-VP1-rv	CCAGGAAGCTCCTCTGTGTC	60°C	1.15	This study
MCPyV-VP1-fw	CACACGGGACCAACTCAAG	60°C	0.98	This study
MCPyV-VP1-rv	AGGTATATCGGGTCCTCTG	60°C	0.98	This study
MCPyV-LT-fw	AAAACACCCAAAAGGCAATG	60°C	1.08	This study
MCPyV-LT-rv	GCAGAGACACTCTTGCCACA	60°C	1.08	This study
SV40-LT-fw	CCGATATCATGGATAAAGTTTTAAACAGAG	55°C	-	This study
SV40-LT-rv	GGGCTCGAGGCAGTGCAGCTTTTTCCTTTG	55°C	-	This study
MCPyV EcoRV_F	CCGATATCATGGATTTAGTCCTAAATAGG	58°C	-	This study
MCPyV_XhoI_R	GGGCTCGAGTTGAGAAAAAGTACCAGAATCT TG	58°C	-	This study
MCPyV-LT-s	ATGGATTTAGTCCTAAATAGGAAAG	56°C	-	Shuda et al., 2009
MCPyV-LT-as	CTCATCAAACATAGAGAAGTCAC	56°C	-	Shuda et al., 2009

[a] Ct values (determined by using the Rotorgene Software version 1.7) were plotted against the log10 value of template concentration and the slope (M) determines the reaction efficiency according to (10-1/M)-1=1.
