# Supplementary material for: Replication, Gene Expression and Particle Production by a Consensus Merkel Cell Polyomavirus (MCPyV) Genome
Source: PLoS One. 2011 Dec 27;6(12):e29112. doi: 10.1371/journal.pone.0029112 (PMC3246459; doi:10.1371/journal.pone.0029112)
Supplement: Table S3 — Accession numbers. (PDF) [file pone.0029112.s009.pdf]

Supplementary Table S3: Accession numbers

strain/isolate	accession #	MCC[a]	MCVSyn[b]	phylog. analysis[c]
MCVSyn	JN707599			*
isolate 06b	HM011538.1			*
isolate 09b	HM0115349.1			*
isolate 10b	HM011540.1			*
isolate 12a	HM011541.1			*
isolate 12b	HM011542.1			*
isolate 13a	HM011543.1			*
isolate 14a	HM011544.1			*
isolate 14b	HM011545.1			*
isolate 15a	HM011546.1			*
isolate 15b	HM011547.1			*
isolate 16b	HM011548.1			*
isolate 17b	HM011549.1			*
isolate 18b	HM011550.1			*
isolate 20b	HM011551.1			*
isolate 25b	HM011552.1			*
isolate 26b	HM011553.1			*
isolate 30b	HM011554.1			*
isolate 85	JF813002.1			*
isolate R30a	HM011557.1			*
isolate R17a	HM011555.1			*
isolate R17b	HM011556.1			*
MCVw156	HM355825.1			*
MCC206	FJ173812.1	*	*	
MCC322	FJ173811.1	*	*	
MCC339	EU375804.1	*	*	*
MCC344	FJ173807.1	*	*	
MCC345	FJ173808.1	*	*	
MCC347	FJ173810.1	*	*	
MCC348	FJ173809.1	*	*	
MCC349	FJ173813.1	*	*	
MCC350	EU375803.1	*	*	*
MCC352	FJ173814.1	*	*	
MCC366	FJ173803.1	*	*	
MCC85	FJ173804.1	*	*	
isolate 344	JF812999.1	*		*
isolate 349	JF813000.1	*		*
isolate 352	JF813001.1	*		*
MKL-1	FJ173815.1	*	*	*
TKS	FJ464337.1	*		*

Entries marked with an asterisk denote:
[a] MCC-derived sequences
[b] sequences used to generate the MCVSyn consensus genome
[c] full-length genomes used for the phylogenetic analyses in figures 1 and S1
